# Expression of a Plastid-Targeted Flavodoxin Decreases Chloroplast Reactive Oxygen Species Accumulation and Delays Senescence in Aging Tobacco Leaves

**DOI:** 10.3389/fpls.2018.01039

**Published:** 2018-07-17

**Authors:** Martín L. Mayta, Anabella F. Lodeyro, Juan J. Guiamet, Vanesa B. Tognetti, Michael Melzer, Mohammad R. Hajirezaei, Néstor Carrillo

**Affiliations:** ^1^Instituto de Biología Molecular y Celular de Rosario (IBR-UNR/CONICET), Facultad de Ciencias Bioquímicas y Farmacéuticas, Universidad Nacional de Rosario, Rosario, Argentina; ^2^Instituto de Fisiología Vegetal (INFIVE–UNLP/CONICET), La Plata, Argentina; ^3^Mendel Centre for Plant Genomics and Proteomics, Central European Institute of Technology, Masaryk University, Brno, Czechia; ^4^Leibniz Institute of Plant Genetics and Crop Plant Research, OT Gatersleben, Seeland, Germany

**Keywords:** senescence, chloroplasts, reactive oxygen species, redox poise, flavodoxin, transgenic plants

## Abstract

Leaf senescence is a concerted physiological process involving controlled degradation of cellular structures and reallocation of breakdown products to other plant organs. It is accompanied by increased production of reactive oxygen species (ROS) that are proposed to signal cell death, although both the origin and the precise role of ROS in the execution of this developmental program are still poorly understood. To investigate the contribution of chloroplast-associated ROS to natural leaf senescence, we used tobacco plants expressing a plastid-targeted flavodoxin, an electron shuttle flavoprotein present in prokaryotes and algae. When expressed in plants, flavodoxin specifically prevents ROS formation in chloroplasts during stress situations. Senescence symptoms were significantly mitigated in these transformants, with decreased accumulation of chloroplastic ROS and differential preservation of chlorophylls, carotenoids, protein contents, cell and chloroplast structures, membrane integrity and cell viability. Flavodoxin also improved maintenance of chlorophyll-protein complexes, photosynthetic electron flow, CO_2_ assimilation, central metabolic routes and levels of bioactive cytokinins and auxins in aging leaves. Delayed induction of senescence-associated genes indicates that the entire genetic program of senescence was affected by flavodoxin. The results suggest that ROS generated in chloroplasts are involved in the regulation of natural leaf senescence.

## Introduction

Leaf senescence is a genetically coordinated process resulting in progressive decay and ultimately death of the tissue, with remobilization of the nutrients to other plant organs such as more apical leaves, seeds and/or storage tissues (Gregersen et al., 2013; Zhang and Zhou, 2013; Schippers et al., 2015). The most conspicuous visual symptom of leaf senescence is yellowing caused by dismantling of the pigment-protein complexes of chloroplasts and degradation of the constituent *Chl*. In many plant species grown with sufficient nutrition, leaf senescence is age-dependent, and tobacco has been extensively used as a model species to study this process (Uzelac et al., 2016, and references therein).

While senescence implies cell death and degradation of cellular components, it is not simply a destructive process but an ordered physiological pathway controlled by a genetic program and affected by environmental factors (Jibran et al., 2013; Ay et al., 2014). To destroy themselves and recycle nutrients, plant cells need to maintain some gene expression and metabolite transport capacity while their macromolecules and cellular structures are being dismantled by some of the new gene products (Ávila-Ospina et al., 2014; Schippers et al., 2015). Genes that were found to be differentially induced during senescence have been termed SAGs, and used as molecular markers of the process. Many products encoded by SAGs are enzymes involved in protein degradation, underscoring the relevance of nitrogen salvage and mobilization during senescence (Uzelac et al., 2016).

How gene expression is regulated in senescing tissues and how senescence starts and proceeds are among the most significant biological questions (Penfold and Buchanan-Wollaston, 2014; Schippers et al., 2015). In addition, senescence may affect crop yield and quality in several ways. As indicated, senescence facilitates carbon and nitrogen remobilization from leaves to grains, fruits, etc., which improves their nutritional value, but it might also reduce crop yield when induced prematurely under environmental stress (Gregersen et al., 2013; Antonietta et al., 2016; Moschen et al., 2016). The photosynthetic capability of leaves declines sharply with the onset of senescence, limiting biomass production, whereas fruit senescence substantially contributes to postharvest losses during transportation and storage (Moschen et al., 2016).

Increased accumulation of ROS, such as hydrogen peroxide (H_2_O_2_), superoxide (O_2_^.-^) and the hydroxyl radical (HO^.^), has been shown to precede and accompany senescence in many organisms including plants (Wu et al., 2012; Xie et al., 2014; Biswas and Mano, 2015; Garapati et al., 2015). ROS can be produced in various cellular compartments but it is unclear how the different sources contribute and/or interact to modulate cell death in senescing tissues. Models of cell death execution are often inspired by animal systems, where mitochondria play a crucial role (Circu and Aw, 2010). Involvement of these organelles in plant senescence processes has also been documented for non-photosynthetic tissues (Van Aken and Van Breusegem, 2015; Rogers and Munné-Bosch, 2016). In leaves, however, chloroplasts are the major source of ROS in the light (Wang and Blumwald, 2014; Noctor and Foyer, 2016). Moreover, disassembly of photosynthetic complexes takes place during leaf aging, leading to faulty distribution and misrouting of reducing equivalents in the photosynthetic electron transport chain (ETC), which is a prime cause of increased ROS propagation in plant cells (Juvany et al., 2013).

Cell death can also be induced during episodes of biotic and abiotic stress (Gepstein and Glick, 2013; Gregersen et al., 2013). Stress situations results in universal down-regulation of Fd, the final electron acceptor of the ETC, which in turns leads to acceptor side limitation and increased ROS propagation in chloroplasts (Tognetti et al., 2006; Pierella Karlusich et al., 2014, 2017). By using tobacco *pfld* lines expressing a plastid-targeted cyanobacterial Fld, we were able to show that chloroplast-generated ROS contribute to localized cell death during various plant-pathogen interactions (Zurbriggen et al., 2009; Rossi et al., 2017). Fld is an electron shuttle flavoprotein isofunctional with Fd, which is found in phototrophic microorganisms, but not in plants (Pierella Karlusich et al., 2015). When expressed in plant chloroplasts, however, Fld can accept reducing equivalents from the ETC, bypassing the limitation imposed by Fd decline and diverting these equivalents from oxygen into productive pathways of the plastid. In doing so, it specifically decreases chloroplast ROS accumulation under adverse situations (Tognetti et al., 2006; Zurbriggen et al., 2009). Leaves from *pfld* lines did not develop local cell death upon inoculation with a non-host microorganism, while tissue destruction was extensive in WT siblings (Zurbriggen et al., 2009). The results suggested that Fld could modulate chloroplast ROS build-up and signaling during other physiological processes of the plant, including senescence.

The aim of this study was therefore to use these transgenic lines to probe the role played by chloroplast ROS during leaf senescence. Analysis of aging leaves showed lower ROS levels in plants expressing plastid-targeted Fld compared to the WT. This effect was accompanied by delayed leaf senescence, as indicated by less protein and pigment degradation, differential preservation of cell structure and viability, maintenance of photosynthetic complexes and activity, and delayed induction of SAGs. Extensive metabolic profiling also showed differential preservation of Suc, sugar phosphates and most amino acids, suggesting that Fld protected central metabolic routes from age-dependent inactivation. In addition, Fld expression delayed senescence induced by salicylic acid (SA). The results indicate that Fld expression affects leaf senescence, likely through modulation of chloroplast ROS, and identify a novel pathway involved in the execution of this critical developmental program.

## Materials and Methods

### Plant Material and Growth Conditions

**FIGURE 1:**
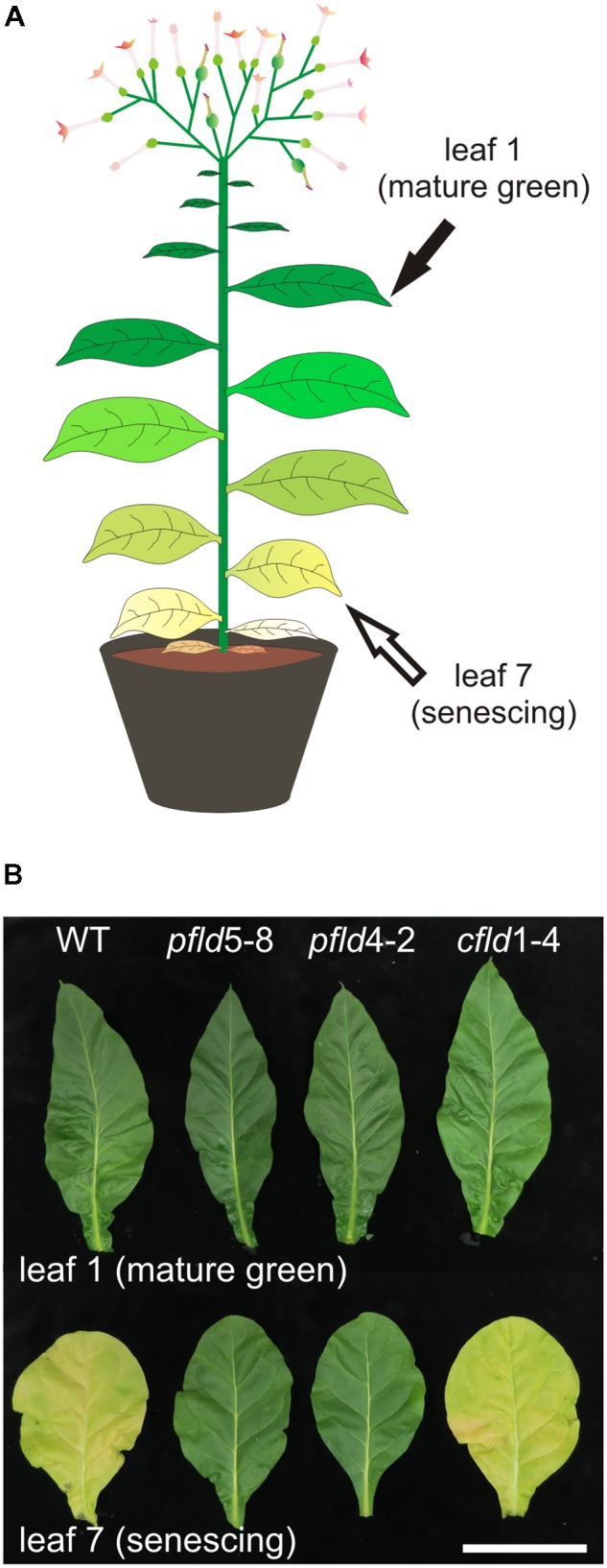
Transgenic tobacco plants expressing plastid-targeted Fld displayed a stay-green phenotype. **(A)** Cartoon depicting a flowering WT tobacco plant at 73 dpg with leaves at different developmental stages. **(B)** Leaves 1 and 7 from WT, *pfld* and *cfld* plants were photographed at 73 dpg. Bar = 10 cm.

Wild-type, *pfld* and *cfld* tobacco plants (*Nicotiana tabacum* cv. Petit Havana) were grown in soil (3-L pots) at a light intensity of 250 μmol photons m^-2^ s^-1^, with a 16-h photoperiod, 20–28°C and a relative humidity of 80% (growth chamber conditions). They had 19–20 fully expanded leaves at the onset of flower blooming [60–63 days post germination (dpg)], and ceased to produce new leaves after flowering. Leaves were numbered at 73 dpg, from the youngest fully expanded leaf downward (**Figure 1A**). Senescence was studied in leaf 1 (mature green) and leaf 7 (senescing) at 73 and 81 dpg. For phenotypic evaluation, leaves and fruits were collected at 83–85 dpg, weighed (fresh weight, FW), and then kept in an oven at 65°C until constant weight (dry weight), which was achieved in 24–48 h.

### Structural Analysis of Leaf Tissue by Light and Transmission Electron Microscopy

Cuttings of 2 mm^2^ from the central part of leaf 1 and 7 from three different plants of WT, *pfld* and *cfld* lines at 73 dpg were used for sample preparation for histological and ultrastructural examination. Conventional and microwave-assisted fixation substitution, resin embedding, sectioning, and microscopic analysis were performed as described (Kraner et al., 2017). For cell area measurements, leaf disks (0.5 cm in diameter) were removed from the interveinal leaf region of 5–9 independent plants and fixed in 96% (v/v) ethanol, followed by incubation in 85% (w/v) lactic acid for clearing.

### *In Situ* Detection of Reactive Oxygen Species

Reactive oxygen species cellular localization was determined by confocal microscopy in an Eclipse TE–2000–E2 Confocal Laser Scanning Microscope (Nikon) with excitation at 488 nm and emission at 515/530 nm, after leaf staining with the ROS-sensitive fluorescent probe DCFDA. Leaf disks (1 cm in diameter) from 6 plants of each line were collected during the light period, vacuum-infiltrated in the dark with 50 μM DCFDA in 10 mM Tris-HCl pH 7.5, incubated in the dark for 1 h in the same solution, washed briefly and mounted in water. Images were acquired with a 20x objective (Plan-Apochromat 20X/0.75), image size 512 × 512 pixels, 16 bit depth. Before recording images, the signal intensity across the entire view was visually inspected in order to prevent signal saturation. Imaging was performed by scanning 7 optical slices (with an interval of ∼1 μm) of the palisade parenchyma immediately next to the epidermis. Fluorescence intensities were estimated using Fiji software (Schindelin et al., 2012). Stacks were compiled to single images (z-projections) and presented as a “sum slices” projection type. Fluorescence intensities were calculated using the z-projections.

### Induction of Senescence in Leaf Disks by Application of Salicylic Acid (SA)

Leaf disks (1 cm in diameter) punched from leaf 1 of 63-dpg WT and *pfld*5-8 plants were incubated with 15 mL of 100 μM SA in 3 mM MES pH 5.8 or the same volume of buffer, and incubated under growth chamber conditions. Images were taken after 6 days of SA treatment and disks were subsequently used for photosynthetic measurements and pigment determination.

### Determination of Photosynthetic Activities

Chlorophyll fluorescence measurements were performed using an FL-1 Pulse-modulated Chlorophyll Fluorometer with an ACT1 Actinic Light Control Module (Qubit Systems). *F*_v_ and *F*_m_ were determined after dark adaptation of the attached leaves for 30 min. Subsequently, leaves were exposed to 200 μmol photons m^-2^ s^-1^ of actinic light, and light-adapted values (*F*′_v_, *F*′_m_) were determined at the end of each actinic light period. For SA-treated disks, only light-adapted values were measured. Photosynthetic parameters (*F*_v_/*F*_m_, ϕ_PSII_, 1 – *qP* and NPQ) were calculated as described by Baker (2008).

Net CO_2_ uptake rates were determined at 200 μmol photons m^-2^ s^-1^ according to Hajirezaei et al. (2002), using a portable photosynthesis system LI-6400XT (LI-COR). The CO_2_ concentration of the air entering the leaf chamber and the leaf temperature were adjusted to 400 μmol mol^-1^ and 20°C, respectively.

### Native Gel Electrophoresis and Mass Spectrometry (MS) Analysis of *Chl*-Protein Complexes

*Chl*-containing complexes were separated by Deriphat^®^-PAGE according to Sárvári and Nyitrai (1994) with minor modifications. Briefly, thylakoid membranes were isolated from leaves 1 and 7 of WT and transgenic plants at 73 dpg according to Guiamet et al. (2002), and solubilized in a 9:1 mixture of *n*-(decyl) β-D-maltoside and SDS, using a 40:1 (w/w) detergent/chlorophyll ratio. Extracts were resolved in 5.5–12% polyacrylamide gradient gels in the presence of the non-ionic detergent Deriphat^®^ 160 (sodium *N*-lauryl-β-iminodipropionate). Following electrophoresis, gels were photographed under visible and UV light (∼302 nm) to visualize *Chl* autofluorescence and/or quenching by PSI and PSII reaction centers. *Chl*-containing bands were excised from the Deriphat gels, digested with trypsin and subjected to MS analysis using the service of the Analytical Biochemistry and Proteomics Unit of the Institut Pasteur of Montevideo (Uruguay). Protein identification by MALDI-TOF-TOF MS (peptide mass fingerprinting, MS/MS ion search) and database search were performed using the same service, as described by Lima et al. (2011). Spectra were acquired on a 4800 MALDI-TOF-TOF mass spectrometer (Applied Biosystems) on positive reflector mode. Proteins were identified as the highest-ranking result by searching in the National Center for Biotechnology Information database (NCBI) using Mascot^1^. For positive identification, the score of the result of [-10 × log(P)] had to be over the significance threshold level (*P* < 0.05). Selected proteins were represented by at least two different peptides in the spectra.

### RNA Isolation and Quantitative Reverse-Transcription (qRT)-PCR Analysis

Total RNA was isolated from 100 mg of tobacco leaves using the TriPure reagent (Sigma–Aldrich), according to the manufacturer’s instructions, and reverse-transcribed with the MMLV enzyme (Invitrogen) and oligo(dT)_12-18_. Real-time PCR reactions were carried out in a Mastercycler^®^ ep *realplex*^4^ thermocycler (Eppendorf) using Platinum Taq DNA polymerase (Invitrogen) and SYBR Green I (Roche) to monitor the synthesis of double-stranded DNA. Relative transcript levels were determined for each sample and normalized against the levels of tobacco elongation factor 1α (EF1α) cDNA (Schmidt and Delaney, 2010). Primers (Supplementary Table S6) were designed using the “Primer3Plus” software^2^ with an annealing temperature of 55°C.

### Measurements of Soluble Sugars, Starch, Amino Acids, and Metabolites

Soluble sugars were determined by enzyme-coupled assays according to Ghaffari et al. (2016). Starch levels were measured in the insoluble fraction of the same extracts used for estimation of soluble sugars. Starch was hydrolyzed by incubation with amyloglucosidase (Sigma–Aldrich) for 16 h at 37°C, and the resulting Glc was assayed as described above.

For amino acid determinations, samples were derivatized using the fluorescing reagent AQC (6-aminoquinolyl-*N*-hydroxysuccinimidylcarbamate). Three mg of home-made AQC (IPK, Germany) were dissolved in 1 ml acetonitrile and incubated for 10 min at 55°C. The reagent was stored at 4°C and used for up to 4 weeks. For derivatization of the sample, 10 μl of the reagent solution were employed for each sample which contained 0.8 ml of 0.2 M boric acid, pH 8.8, and 10 μl of extract. Separation of soluble amino acids was performed by a newly developed method using ultra pressure reversed-phase chromatography (UPLC) AcQuity H-Class (Waters). The UPLC system consisted of a quaternary solvent manager, a sample manager-FTN, a column manager and a fluorescent detector (PDA eλ Detector). Separation was carried out on a C18 reversed-phase column (ACCQ Tag Ultra C18, 1.7 μm, 2.1 × 100 mm) with a flow rate of 0.7 ml per min and a duration of 10.2 min. The column was heated at 50°C during the whole run. Detection wavelengths were 266 nm for excitation and 473 nm for emission. The gradient was accomplished with four solutions prepared from two different buffers: eluent A concentrate and eluent B for amino acid analysis (Waters). Eluent A was pure concentrate, eluent B was a mixture of 90% LCMS water (Chemsolute) and 10% eluent B concentrate, eluent C was pure eluent B concentrate and eluent D was LCMS water. The column was equilibrated with a mixture of eluent A (10%) and eluent C (90%) for at least 30 min. The gradient was generated as follows: 0 min, 10% A and 90% C; 0.29 min, 9.9% A and 90.1% C; 5.49 min, 9% A, 80% B and 11% C; 7.1 min, 8% A, 15.6% B, 57.9% C and 18.5% D; 7.3 min, 8% A, 15.6% B, 57.9% C and 18.5% D; 7.69 min, 7.8% A, 70.9% C and 21.3% D; 7.99 min; 4% A, 36.3% C and 59.7% D; 8.68 min, 10% A, 90% C; and 10.2 min, 10% A and 90% C.

Metabolite separation and detection were performed according to Ghaffari et al. (2016), using an ion chromatography system (Dionex Thermofisher) connected to a triple quadrupole mass spectrometer QQQ6490 (Agilent Technologies). ESI-MS/MS analysis was conducted as described by Ghaffari et al. (2016).

### Phytohormone Analysis

Endogenous CKs and auxins were extracted from leaf tissue and analyzed essentially as described by Rasmussen et al. (2014). An Agilent 1290 Infinity system connected to an Agilent triple quadrupole mass spectrometer QQQ6490 was used for separation and detection of individual hormones. Separated compounds were ionized at atmospheric pressure via electrospray and directed to the mass spectrometer. The control of the complete system and recording of the spectra were performed with the MassHunter, release B.04.00 (B4038). Separation of hormones was performed on an Eclipse Plus C18 column, RPHD 1.8 μm, 2.1 × 50 mm. The gradient was accomplished with 0.1% (v/v) formic acid in LCMS grade water as buffer A and 0.1% (v/v) formic acid in LCMS grade methanol (Chemsolute) as buffer B. The column was equilibrated with a mixture of buffer A (86.5%) and B (13.5%) at a flow rate of 0.4 ml min^-1^ and heated at 40°C during the whole measurement. The gradient was produced by changes of buffer B as follows: 0–5 min at 18%, 5–6 min at 70%, 6–7 min at 99%, 7–7.1 min at 13.5%, and kept up to 9 min at 13.5%. The whole duration of the run was 9.0 min.

### Analytical Procedures

Chlorophylls and *Car* were determined spectrophotometrically after extraction with 96% (v/v) ethanol (Lichtenthaler, 1987). Total soluble protein concentrations were measured in cleared leaf extracts by the Bradford (1976) procedure, using bovine serum albumin as standard. The levels of various individual leaf proteins were estimated by SDS–PAGE and immunoblotting. Extracts were resolved on 15% polyacrylamide gels, transferred to nitrocellulose membranes and decorated with polyclonal antibodies raised against tobacco Rubisco and Fd, and tomato glutamine synthetase 2 (GS2). To facilitate visualization of declining protein levels in aging leaves, higher amounts of extract were loaded for leaf 7, relative to leaf 1, for total protein, Rubisco and Fd determinations.

Cell death in tobacco leaf tissue was monitored by Evans Blue staining essentially as described by Goupil et al. (2012). Briefly, leaf disks (1 cm in diameter) from 4 to 5 plants from each line and age (two disks per leaf) were infiltrated twice with a 0.25% (w/v) Evans Blue (Sigma–Aldrich) solution and incubated for 30 min at 25°C in a rotary shaker, rinsed extensively to remove the excess dye and ground in a tissue grind tube with 1 ml of 1% (w/v) SDS. The suspension was centrifuged for 20 min at 20,000 *g*, and the supernatant was used for dye quantification by monitoring the absorbance at 600 and 680 nm. Cell death was expressed as A_600_/A_680_ ratios.

Electrolyte leakage was evaluated by placing leaf disks in 1 ml of distilled water and measuring the increase in conductance of the medium using a B-173 conductivity meter (Horiba), essentially as described by Tognetti et al. (2006). Two incubation times (5 and 17 h) were assayed to confirm the leakage trend.

Leaf cell viabilities were determined by measuring the reduction of the dehydrogenase substrate TTC (Sigma–Aldrich) in cleared extracts, according to Steponkus and Lanphear (1967). Viabilities were expressed as the absorbance change per g FW.

### Statistical Analyses

Data were analyzed using one-way ANOVA and Holm-Sidak or Duncan multiple range tests. When the normality and/or equal variance assumptions were not met, Kruskall–Wallis one-way ANOVA between lines, and Dunn’s multiple range tests were used. Unless otherwise stated, significant differences refer to statistical significance at *P* < 0.05.

## Results

### Expression of Plastid-Targeted Fld Delayed Leaf Senescence in Transgenic Tobacco Plants

Cell death and ROS accumulation during leaf senescence were studied in tobacco *pfld* lines, using WT plants and a transformant in which Fld was targeted to the cytosol (*cfld*1-4 line) as controls (Tognetti et al., 2006). Independent lines *pfld*5-8 and *pfld*4-2 accumulate 60–70 pmol Fld g^-1^ leaf FW in chloroplasts, equivalent to those of its endogenous isofunctional counterpart Fd, and display similar levels of protection against biotic and abiotic stresses (Tognetti et al., 2006; Zurbriggen et al., 2009; Rossi et al., 2017).

When cultivated under growth chamber conditions (see Materials and Methods), plants from WT, *pfld* and *cfld* lines had similar numbers of leaves and nodes (Supplementary Figure S1 and Supplementary Table S1), and blossomed at about the same time, 60–63 days post germination (dpg). By 73 dpg, the basal leaves from WT and *cfld*1-4 plants were already senescent (Supplementary Figure S1). The youngest fully expanded leaf of plants at 73 dpg was termed leaf 1, and the following leaves numbered from the younger to the older (**Figure 1A**), as is customary in senescence studies using tobacco plants (Golczyk et al., 2014; Almoguera et al., 2015; Uzelac et al., 2016). Since apical growth ceased at the onset of flowering, leaf numbers and positions did not change further (Supplementary Figure S1).

Leaf 1 of plants from all lines looked green and healthy at 73 dpg (**Figure 1B**). *Chl* and *Car* levels of *pfld* leaves were 30–50% higher than those found in WT and *cfld*1-4 siblings (Supplementary Table S1), in agreement with previous reports (Tognetti et al., 2006; Ceccoli et al., 2012). Microscopic evaluation showed that *pfld* leaves contained smaller and more densely packed cells (Supplementary Figures S2A,B), resulting in higher cell numbers per leaf cross-section compared to *cfld*1-4 and WT counterparts (Supplementary Figure S2C). This change in leaf tissue organization did not significantly affect leaf biomass (Supplementary Table S1), but could explain the elevated pigment contents per leaf cross-section observed in the transformants. The average number and dry weight of fruits were also similar in the four lines analyzed (Supplementary Table S1).

**FIGURE 2:**
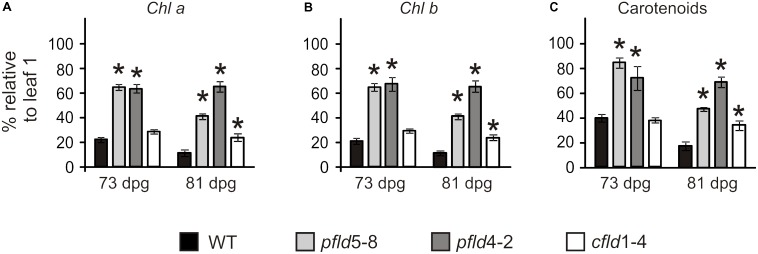
Fld expression in plastids delayed degradation of *Chl a* **(A)**, *Chl b* **(B)**, and carotenoids **(C)** during leaf senescence. Plants were assayed at 73 and 81 dpg. Pigment levels are expressed as the percentage of the corresponding values determined in leaf 1 of the same plant. Data shown are means ± SE (*n* = 4–6). Asterisks indicate significant differences with respect to the wild type (ANOVA, *P* < 0.05).

Visual symptoms of senescence, in the form of yellowing and loss of turgor, were evident in leaf 7 of WT and *cfld*1-4 lines at 73 dpg, whereas the equivalent leaves of *pfld* plants remained green (**Figure 1B**). To rule out the possibility that color retention in these leaves simply reflected their higher pigment contents per area, *Chl* and *Car* levels were determined in leaf 7 and referred to those measured in leaf 1 of the same line and plant. As shown in **Figure 2**, leaf 7 of WT and *cfld*1-4 plants had 20–30% *Chl* and less than 40% *Car* relative to leaf 1 by 73 dpg, and even lower values for both types of pigment at 81 dpg. Their *pfld* counterparts, instead, still retained a significant fraction of *Chl* (40–70%; **Figures 2A,B**) and *Car* (50–80%; **Figure 2C**). While **Figure 2** shows results obtained with leaves 1 and 7, differential pigment preservation in plants expressing Fld in chloroplasts was also evident in the intermediate leaves (Supplementary Figure S3).

Accelerated senescence can be induced by certain hormones and a number of stress treatments including darkness. Among hormones, SA has been shown to participate in senescence regulation (Rivas-San Vicente and Plasencia, 2011). Indeed, Arabidopsis mutants blocked in SA catabolism exhibited an early senescing phenotype, whereas the down-regulation of hormone levels led to delayed senescence (Zhang et al., 2013). In line with those observations, exposure of WT leaf disks to 100 μM SA caused significant declines in *Chl* contents after 6 days of treatment (Supplementary Figures S4A,B). The quantum yield of PSII (Φ_PSII_), which provides an estimation of the electron flow through PSII and hence, of photosynthetic electron transport (Baker, 2008), was also strongly decreased by incubation with the hormone (Supplementary Figure S4C). Expression of plastid-targeted Fld resulted in virtually complete reversion of the SA effects (Supplementary Figure S4).

The results indicate that age-associated pigment degradation was alleviated in *pfld* plants, and that this effect was not a simple outcome of the changes in leaf cell packing and pigment content per leaf area introduced by Fld presence. This very interesting finding on the relationship between chloroplast Fld expression and leaf development certainly deserves a more detailed investigation, but is beyond the scope of the present article and will not be addressed further.

### Plastid-Targeted Fld Decreased ROS Build-Up in Aging Leaves of Transgenic Tobacco Plants

**FIGURE 3:**
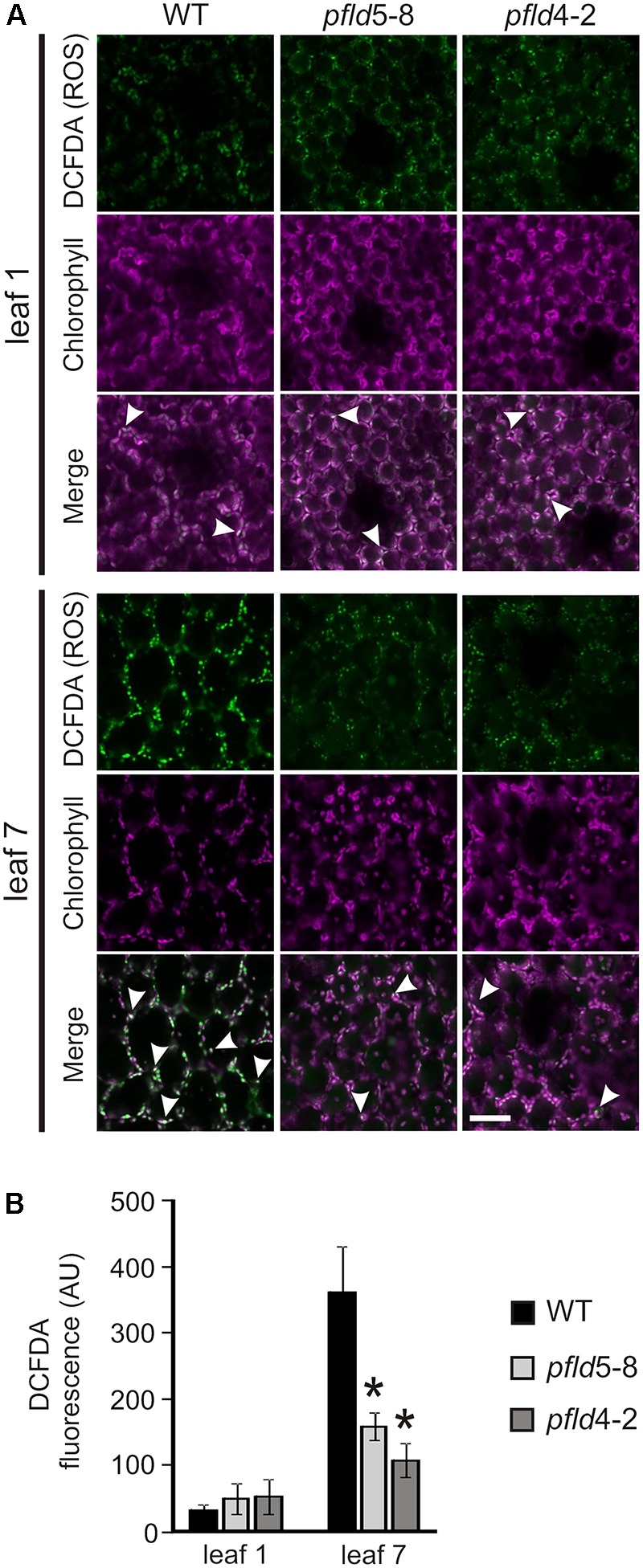
Expression of a plastid-targeted Fld in transgenic tobacco suppressed ROS build-up in chloroplasts of aging leaves. **(A)** Fluorescent detection of oxidant formation in leaves was performed using the ROS-sensitive probe DCFDA as described in Material and Methods. Bar = 40 μm. ROS (green), chlorophyll (magenta) and merge images are shown. Arrowheads show merge of *Chl* and ROS-derived signals in chloroplasts. **(B)** ROS-dependent fluorescence was quantified on multiple image stacks with the Image J software. Data shown are means ± SE of 4-6 biological replicates. Fluorescence intensities are expressed in arbitrary units (AU). Asterisks indicate values significantly different from the wild type (ANOVA, *P* < 0.05). Plants were assayed at 81 dpg.

To determine if the “*SGR*” phenotype displayed by *pfld* lines correlated with lower ROS generation, leaves of WT, *pfld*5-8 and *pfld*4-2 plants were treated with the ROS-sensitive fluorescent probe DCFDA for visualization by confocal laser scanning microscopy. Similar results were obtained at 73 and 81 dpg, and typical images obtained with leaves from the older plants are thus shown in **Figure 3A**. Most of the label was associated to chloroplasts, co-localizing with *Chl* auto-fluorescence (**Figure 3A** and Supplementary Figure S5). Image analysis indicates that ROS-dependent fluorescence was low and similar in leaf 1 of the four lines (**Figure 3**). It increased 11.5-fold in leaf 7 of WT plants compared to leaf 1, but less than 3-fold in their *pfld* counterparts (**Figure 3B**). Indeed, ROS levels in leaf 7 of *pfld* plants were only 35–45% of those found in the wild type (**Figure 3B**). The results indicate that ROS build-up in chloroplasts of aging leaves was largely prevented in plants expressing a plastid-targeted Fld, in agreement with the antioxidant role exhibited by this flavoprotein under abiotic and biotic stresses (Tognetti et al., 2006; Zurbriggen et al., 2009; Rossi et al., 2017).

### Senescence Delay Was Accompanied by Preservation of Cell Structure and Viability in *pfld* Leaves

**FIGURE 4:**
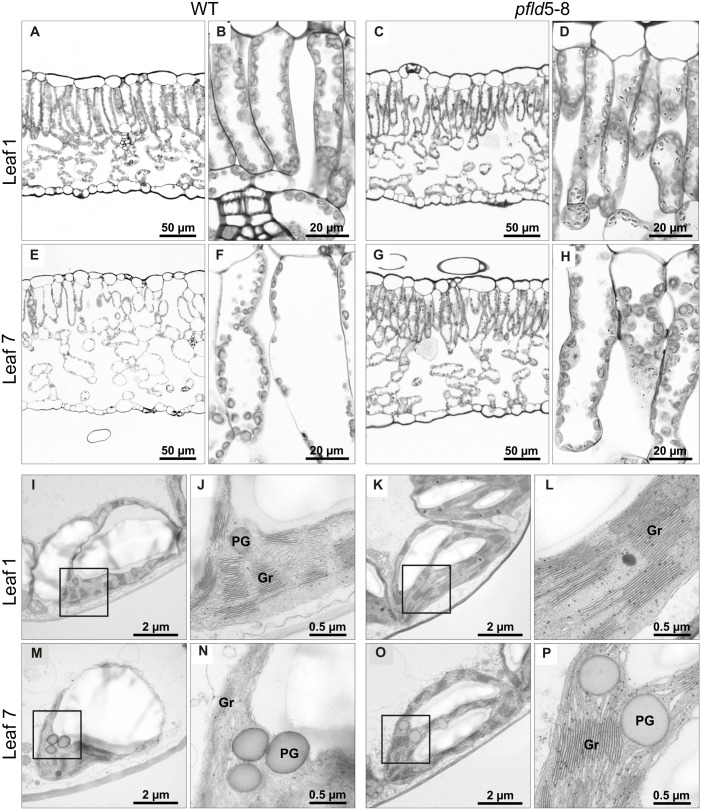
Fld expression in plastids prevented loss of tissue and chloroplast integrity during leaf senescence. Structural analysis of leaf cells and chloroplasts in cross-sections of leaves 1 and 7 from WT and *pfld*5-8 plants at 73 dpg. Light microscopic images **(A–H)**, and TEM micrographs **(I–P)** of palisade parenchyma tissue showing cells and chloroplasts at two different magnifications. Gr, grana; PG, plastoglobuli.

Symptoms of leaf senescence were reflected in the structure of cells and plastids. For all lines, mesophyll tissue from leaf 1 of tobacco plants at 73 dpg contained a single palisade layer of cylindrical cells and an abaxial region of spongy parenchyma with less densely packed cells (**Figures 4A–D** and Supplementary Figures S6A–D). Both cell types exhibited a large central vacuole surrounded by peripheral cytoplasm rich in chloroplasts (**Figures 4B,D** and Supplementary Figures S6B,D). Senescing leaf 7 from WT and *cfld*1-4 plants was characterized by a significant disorganization of cells in the palisade and spongy parenchyma, with prominent intercellular spaces among them (**Figures 4E,F** and Supplementary Figures S6E,F). In contrast, both cell layers appeared well structured and organized in leaf 7 of *pfld* plants (**Figures 4G,H** and Supplementary Figures S6G,H).

Chloroplast ultrastructure, as determined by transmission electron microscopy (TEM), showed typical thylakoid organization in leaf 1 of all lines (**Figures 4I–L** and Supplementary Figures S6I–L). Grana stacking appeared disrupted in chloroplasts from WT and *cfld*1-4 leaf 7, with visible dilatation of the thylakoid membranes and accumulation of plastoglobuli, presumably derived from membrane degradation as a typical feature of senescence (**Figures 4M,N** and Supplementary Figures S6M,N). Chloroplasts from the equivalent leaf of *pfld* plants displayed instead normal structures and resembled those of mature green leaves (**Figures 4O,P** and Supplementary Figures S6O,P). Although plastoglobuli could also be found in these plastids, they were significantly fewer and smaller compared to WT and *cfld*1-4 chloroplasts.

To determine if preservation of cell and chloroplast structures correlated with improved viability, several parameters associated with cell integrity were evaluated. Supplementary Figure S7A shows that cell death, as estimated by the Evans Blue staining assay (Baker and Mock, 1994), was reduced by 50–70% in leaf 7 of *pfld* plants at 73 dpg, relative to the equivalent leaves from WT and *cfld*1-4 siblings. In line with these observations, membrane integrity was differentially preserved in leaf 7 of *pfld* plants, as indicated by measurements of electrolyte leakage (Supplementary Figure S7B).

Cell viability was also estimated in cleared soluble extracts by measuring their ability to reduce the broad-range dehydrogenase substrate TTC, a widely employed biochemical marker of metabolic activity (Bernstein et al., 2010). The TTC reduction activity declined ∼80% in leaf 7 of WT and *cfld*1-4 plants relative to leaf 1, but only 40–50% in siblings expressing plastid-targeted Fld (Supplementary Figure S7C).

The results indicate that the presence of Fld, and the concomitant decline of ROS levels in chloroplasts, helped to decrease oxidative damage and maintain viability in aging leaf tissues.

### Fld Expression in Plastids Prevented Senescence-Associated Protein Degradation

**FIGURE 5:**
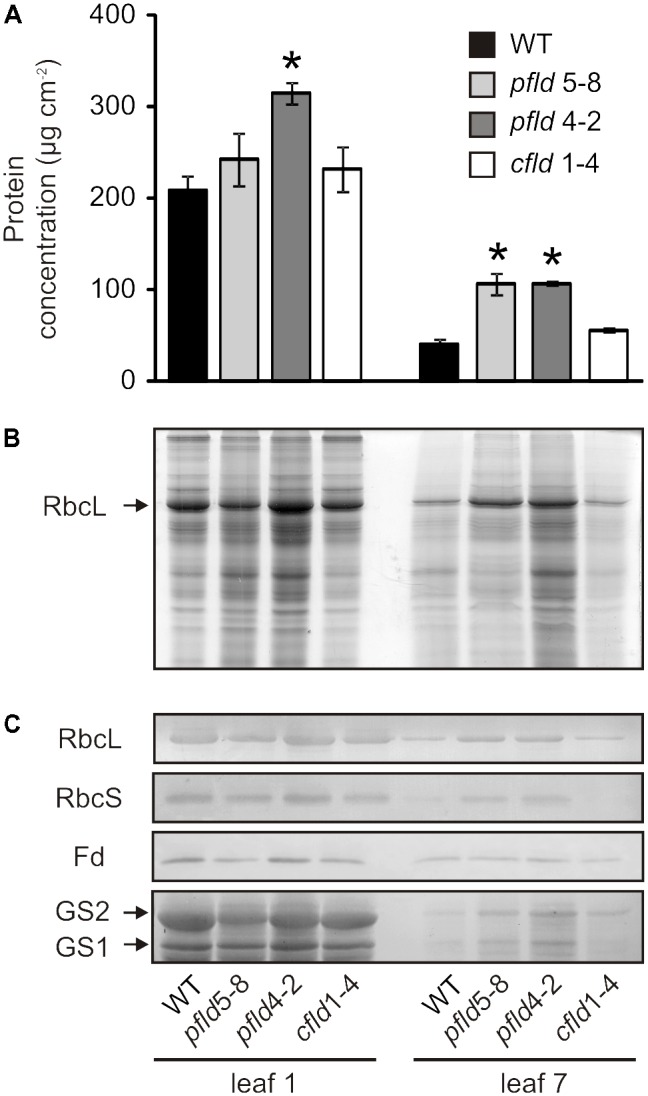
Plastid-targeted Fld delayed protein degradation during leaf senescence. **(A)** Total protein concentrations in cleared leaf extracts. Values are means ± SE (*n* = 3). Asterisks indicate statistically significant differences with respect to the wild type (ANOVA, *P* < 0.05). **(B)** Coomassie Brilliant Blue stain of an SDS-PAGE of leaf extracts. Lysates corresponding to 7.6 or 19 mm^2^ of leaf area were loaded for leaves 1 and 7, respectively. **(C)** Immunoblots of Rubisco large (RbcL) and small subunits (RbcS), ferredoxin (Fd), plastidic glutamine synthetase (GS2) and cytosolic glutamine synthetase (GS1). Soluble proteins contained in 9.5 or 28.5 mm^2^ of foliar area were used for leaves 1 and 7, respectively, in anti-RbcL, anti-RbcS and anti-Fd immunoblots. In the anti-GS immunoblot, cleared extracts corresponding to 57 mm^2^ of leaf area were loaded in each lane. Plants were assayed at 73 dpg. Higher amounts of leaf 7 extracts were loaded to allow visualization of the differences between lines.

SDS-PAGE and immunoblot experiments were used to determine the effect of plastid-targeted Fld on protein degradation as a common feature of leaf senescence. Total soluble protein per unit area decreased more than 80% in leaf 7 of WT and *cfld*1-4 plants at 73 dpg, relative to leaf 1, but only 50–60% in their *pfld* siblings (**Figure 5A**). This effect was largely accounted for by a decrease in Rubisco, as indicated by SDS-PAGE stained for protein (**Figure 5B**), and confirmed by immunoblot experiments (**Figure 5C**). Decline of other leaf components such as the two isoforms of glutamine synthetase was also partially prevented by Fld presence in chloroplasts (**Figure 5C**). Age-dependent down-regulation of Fd, the endogenous Fld counterpart, was instead not affected by expression of the flavoprotein (**Figure 5C**).

### Photosynthetic Activities Were Differentially Preserved in Aging Leaves of Tobacco Plants Expressing Plastid-Targeted Fld

**FIGURE 6:**
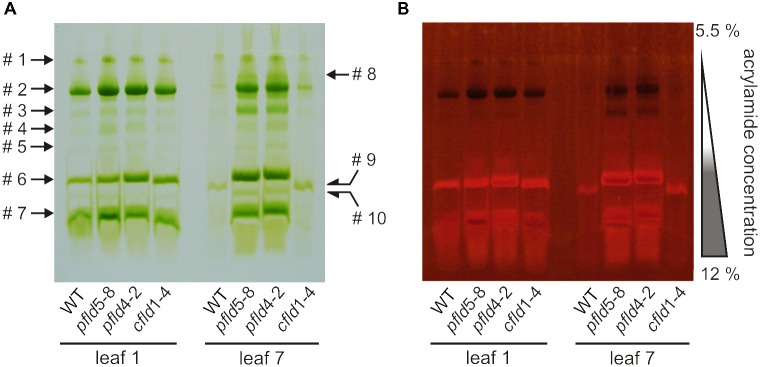
Degradation of *Chl*-protein complexes during senescence was retarded in *pfld* plants. The Deriphat-PAGE system was employed to resolve *Chl*-protein complexes of thylakoid membranes isolated from leaves 1 and 7 of WT, *pfld* and *cfld* plants at 73 dpg. The native green gel was photographed under visible **(A)** and UV light of 302 nm **(B)**. Bands containing antenna not attached to reaction centers fluoresce brightly, while PSI and PSII centers act as sinks for the excitation energy and are therefore non-fluorescent. Soluble extracts corresponding to 20 mm^2^ of leaf area were loaded for leaf 1 of all lines, whereas 50 mm^2^ (WT, *cfld*1-4) or 40 mm^2^ (*pfld* lines) were employed for leaf 7.

Dismantling of *Chl*-protein complexes and inhibition of photosynthetic activity are typical landmarks of plant senescence (Talla et al., 2016, and references therein). To study the fate of *Chl*-containing complexes during leaf aging we performed non-denaturing green gel electrophoresis using the Deriphat^®^-PAGE system (Sárvári and Nyitrai, 1994). Thylakoids isolated from leaf 1 of plants at 73 dpg yielded essentially the same electrophoretic patterns for all lines: three distinct major bands (#2, #6, and #7) and at least four minor ones (#1, #3, #4, and #5) above the threshold of detection and/or resolution (**Figure 6A**).

The composition of the three major bands was analyzed by MS to allow identification of the complexes (Supplementary Table S2). Band #2 contained core components of PSI (P700 apoproteins) and PSII (CP47 and CP43). Quenching of *Chl* fluorescence under UV irradiation confirmed the presence of intact reaction centers in this band (**Figure 6B**). Sárvári and Nyitrai (1994) have reported that PSI and PSII cores migrate close to each other in Deriphat^®^ gels, suggesting that they were not resolved in our experimental conditions, and that band #2 might represent a combination of both. Similar results were obtained by Nath et al. (2013) using Blue Native PAGE in the presence of *n*-(dodecyl) β-D-maltoside.

Bands #6 and #7 contained components of the light-harvesting complex of PSII (LHCII) such as CAB50 (a member of the *Lhcb1* gene family), CP29 and CP26, with band #7 also harboring subunits of the oxygen-evolving complex of PSII (Supplementary Table S2). The amounts of band #1 were too low to be analyzed by MS. Comparison with the results of Sárvári and Nyitrai (1994) suggests that it could represent intact PSI-LHCI complexes. Indeed, resolution of this band by second-dimension SDS-PAGE rendered major components of 110 and 64 kDa, compatible with PSI (Supplementary Figure S8).

Most *Chl*-containing complexes had disappeared in senescing leaf 7 of the same WT and *cfld*1-4 plants (**Figure 6A**). Traces of bands #1 and #2 were barely detectable, whereas a new band (#9), displaying a slightly higher electrophoretic mobility than that of band #6, was apparent (**Figure 6A**). MS analysis indicated that it was most likely a degradation product of the LHCII present in band #6 (Supplementary Table S2). Extracts from leaf 7 of *pfld* plants showed instead a nearly complete preservation of major and minor bands (**Figure 6A**). Band #3 was enhanced, and both *Chl* autofluorescence (**Figure 6B**) and MS results (Supplementary Table S2) identified it as a core PSI, presumably derived from band #2. A new band (#10) migrating ahead of band #6 contained instead components of PSII and LHCII, suggesting that band #2 partially dissociated into band #3 and #10. Still an additional new band (#8) was detected, migrating slightly behind band #2. It is likely that this band corresponds to partially dissociated PSI-LHCI (band #1), but its identity could not be confirmed by MS. The results indicate that the integrity of pigment-protein complexes was maintained for longer periods in *pfld* plants, in line with the observed delay in senescence.

**FIGURE 7:**
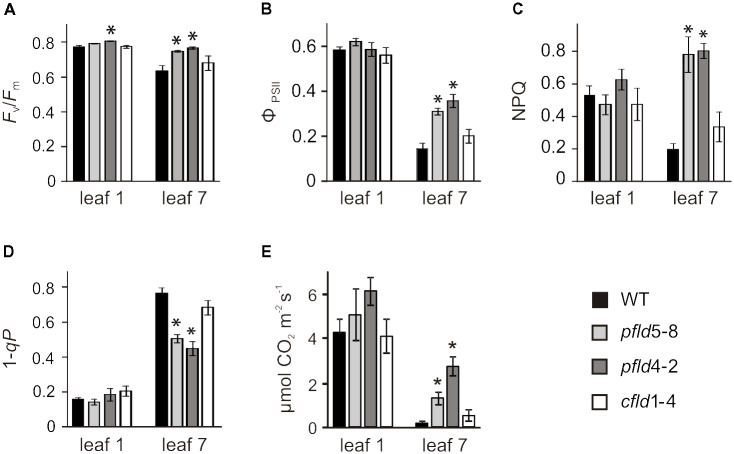
Fld expression in plastids partially prevented loss of photosynthetic activities during leaf senescence. **(A)** Maximum quantum efficiency of PSII photochemistry (*F*_v_/*F*_m_). Plants were dark-adapted for 30 min prior to fluorescence determinations. Measurements of PSII quantum yield (ϕ_PSII_, **B**), non-photochemical quenching (NPQ, **C**), excitation pressure (1 – *qP*, **D**) and CO_2_ assimilation rates **(E)** were carried out at 200 μmol photons m^-2^ s^-1^ as described in section “Material and Methods.” Plants were assayed at 73 dpg. Data reported are means ± SE (*n* = 4–6). Asterisks indicate values significantly different from WT leaves (ANOVA, *P* < 0.05).

To determine how protection of *Chl*-containing antennae and reaction centers in *pfld* leaves affected photosynthetic activity, we carried out *Chl* fluorescence measurements on dark-adapted leaves. The *F*_v_/*F*_m_ ratio, which is customarily used to monitor photodamage to PSII (Baker, 2008), showed a moderate decrease in leaf 7 of WT and *cfld*1-4 plants, relative to leaf 1, whereas damage to PSII was negligible in the equivalent leaf of *pfld* plants (**Figure 7A**). The quantum yield of PSII (Φ_PSII_) also declined 70–80% in leaf 7 of WT and *cfld*1-4 plants compared to leaf 1, but only 40–50% in *pfld* siblings (**Figure 7B**). Values of non-photochemical quenching (NPQ), which reflects the ability of the ETC to dissipate light energy into various processes, and 1 – *qP*, which estimates the fraction of closed PSII reaction centers and the availability of downstream electron acceptors, were similar for leaf 1 of all lines (**Figures 7C,D**). NPQ was ∼60% lower in leaf 7 of WT and *cfld*1-4 plants, indicating that they have impaired ability to remove the excess of excitation energy within chlorophyll-containing complexes. In contrast, leaf 7 of *pfld* plants showed higher NPQ values (**Figure 7C**), which should provide improved tolerance to photoinhibition. The 1 – *qP* parameter increased in leaf 7 of all lines, but significantly less in *pfld* plants, indicating more open PSII reaction centers in the presence of plastid-targeted Fld (**Figure 7D**).

Gas exchange measurements showed that CO_2_ fixation rates per area declined in leaf 7 of all genotypes relative to leaf 1 (**Figure 7E**). Plants expressing plastid-targeted Fld maintained 6- to 8-fold more activity than their WT and *cfld*1-4 siblings (**Figure 7E**), which is consistent with preservation of photosynthetic complexes, Rubisco levels and an increased proportion of open PSII centers in *pfld* leaves.

### Plastid-Targeted Fld Inhibited Age-Associated Changes in Central Metabolic Routes

**FIGURE 8:**
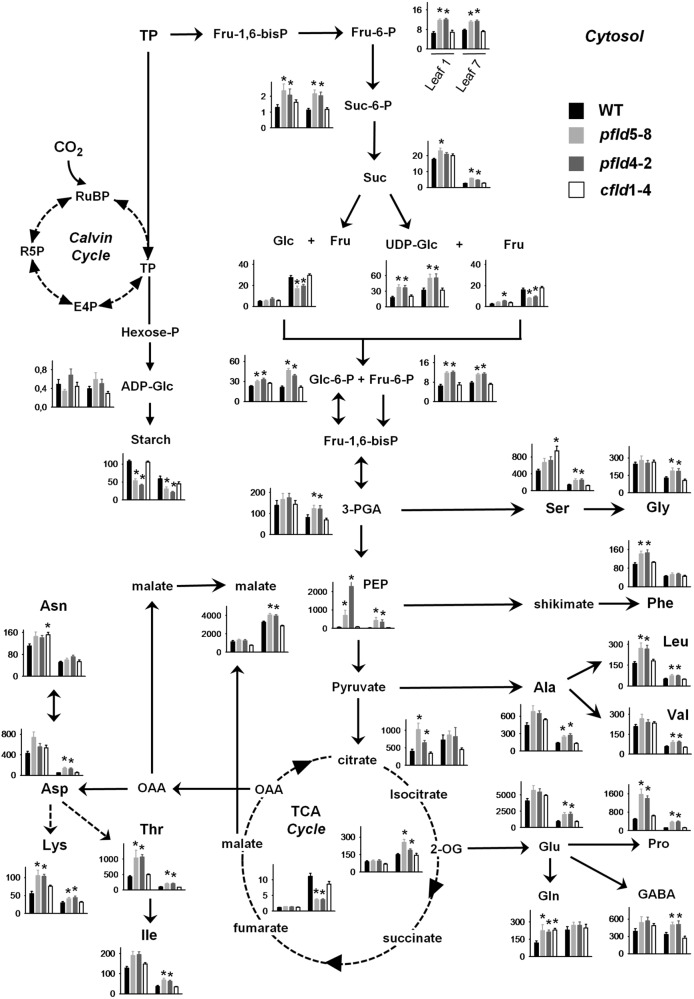
Plastid-targeted Fld protected central metabolic routes in aging leaves. Extracts were prepared from leaves 1 and 7 of WT, *pfld* and *cfld* plants at 73 dpg, and the indicated metabolites were measured as described in section “Material and Methods.” Contents are given as means ± SE (*n* = 8–10). Asterisks indicate statistically significant differences with respect to the wild type (ANOVA, *P* < 0.05). Units in the ordinates are nmol g^-1^ FW, except for starch, Suc, Glc, and Fru, which are expressed in μmol g^-1^ FW. E4P, erythrose-4-phosphate; Fru-6-P, Fru-6-phosphate; Fru-1,6-bisP; Fru-6-P, Fru-1,6-bisphosphate; GABA, γ-aminobutyric acid; Glc-6-P, Glc-6-phosphate; Hexose-P; hexose phosphate; OAA, oxaloacetic acid; 2-OG, 2-oxoglutarate; R5P, ribose-5-phosphate; RuBP, ribulose-1,5-bisphosphate; Suc-6-P, Suc-6-phosphate; TP, triose phosphate.

To determine if the higher CO_2_ assimilation rates of *pfld* plants had an influence on the accumulation of photoassimilates, the levels of several metabolites were measured in leaves 1 and 7 of the four lines at 73 dpg. Results are summarized in **Figure 8**, and the corresponding quantitative data provided in Supplementary Tables S3, S4.

Triose phosphates originating in the Calvin cycle can be used to synthesize starch in chloroplasts, or exported to the cytosol to be converted there into Suc. Levels of Fru-6-phosphate and Suc-6-phosphate, intermediates of Suc synthesis, did not change significantly with the age of the leaf (**Figure 8**). They were higher in *pfld* plants, with contents of the two sugar phosphates in leaf 7 well above those found in their WT and *cfld*1-4 counterparts (**Figure 8**). Unlike those metabolic intermediates, Suc levels decreased strongly in leaf 7 relative to leaf 1. While the decline affected all lines, Suc was still higher in *pfld* leaves (**Figure 8**).

Suc can be cleaved in the cytosol by invertases to yield Fru and Glc, or by Suc synthases to render Fru and UDP-Glc (**Figure 8**). Increases of Glc and Fru levels in leaf 7 of all lines paralleled age-dependent Suc decline. The *pfld* leaves, which retained more Suc, exhibited lower contents of both monosaccharides (**Figure 8**). The 6-phosphate derivatives of Glc and Fru can enter the glycolytic pathway and generate trioses such as 3-phospho-glycerate, PEP and pyruvate for amino acid synthesis in chloroplasts and the tricarboxylic acid (TCA) pathway in mitochondria. Levels of UDP-Glc, sugar phosphates, 3-phospho-glycerate and PEP were significantly higher in *pfld* leaves compared to the wild type (**Figure 8**). Aging affected the accumulation of these intermediates in different ways. UDP-Glc was increased in leaf 7 relative to leaf 1, Glc-6-phosphate was unchanged, and both 3-phospho-glycerate and PEP were drastically diminished (**Figure 8**).

Starch is synthesized in chloroplasts via ADP-Glc, which exhibited accumulation patterns similar to those of UDP-Glc and sugar phosphates (**Figure 8**). Starch displayed a behavior different from that of Suc, with significantly lower levels in *pfld* plants relative to WT and *cfld*1-4 siblings (**Figure 8**). Aging led to starch decreases in leaf 7 of the four lines, but the differences between *pfld* and WT plants were still evident (**Figure 8**).

Levels of the TCA cycle intermediates citrate, 2-oxoglutarate, fumarate and malate increased in leaf 7 relative to leaf 1, but they displayed different patterns depending on the genotype. Fld presence in chloroplasts had only a moderate effect on the contents of citrate (**Figure 8**). Levels of 2-oxoglutarate were higher in leaf 7 of *pfld* plants relative to WT and *cfld*1-4 counterparts (**Figure 8**). Fumarate exhibited a different behavior. It accumulated to low levels compared to the other TCA cycle intermediates, and its age-dependent increase observed in leaf 7 was significantly impaired in plants expressing plastid-targeted Fld. Malate is synthesized in mitochondria by hydration of fumarate mediated by fumarase, and in chloroplasts by sequential action of PEP carboxylase and malate dehydrogenase (Nimmo, 2003). It accumulated to high levels in leaf 7, especially in *pfld* plants (**Figure 8**). Chloroplast malate dehydrogenase is redox-regulated via Trx, and Fld has been shown to act as electron donor for Trx reduction (Tognetti et al., 2006). Then, Fld-driven and Trx-dependent activation of malate dehydrogenase in leaf 7 could account for the higher content of this metabolite.

As indicated previously, trioses and intermediates of the TCA cycle can be used as precursors for amino acid synthesis (**Figure 8**). Virtually all measured amino acids, including γ-aminobutyric acid, declined with age in WT leaves. The only remarkable increase was that of Gln, the canonical amino acid used for nitrogen transport. With few exceptions (His, Asn, Phe), age-dependent amino acid decrease was mitigated by Fld expression in chloroplasts. Among the four amino acids involved in the early steps of nitrogen assimilation (Glu, Gln, Asp, and Asn), the carboxylic acids declined significantly more than the corresponding amides in WT leaves. As a consequence, the Asn/Asp and Gln/Glu ratios increased several-fold in leaf 7 of WT and *cfld*1-4 plants (Supplementary Figures S9A,B), in line with the roles of Gln and Asn in nitrogen mobilization during leaf aging (Havé et al., 2016). In *pfld* leaves these ratios were consistently lower and closer to those found in leaf 1 (Supplementary Figures S9A,B), reflecting the *SGR* phenotype of these transformants. The Gly/Ser ratio, which provides a measure of photorespiration (Wingler et al., 2000), also increased with the age of the leaf in the wild type (Supplementary Figure S9C), indicating higher photorespiratory activity in senescing tissues (Watanabe et al., 2013; Li et al., 2016). This increase was partially prevented by expression of a plastid-targeted Fld in *pfld* plants (Supplementary Figure S9C).

Significant differences in the levels of several metabolites, including starch, sugar phosphates and some amino acids, were observed in leaf 1 of *pfld* plants with respect to the corresponding leaves of WT and *cfld* lines (**Figure 8**). It is likely that the higher cell density illustrated in Supplementary Figure S2 for plants expressing a plastid-targeted Fld accounts for these differences.

### Chloroplast Fld Presence Affected the Levels of Endogenous Cytokinins and Auxins

Cytokinin are reported to retard senescence in plants and detached leaves (Talla et al., 2016), and therefore these hormones have been usually regarded as anti-senescence factors. We measured the major bioactive CK species synthesized through the mevalonate pathway, *cis*-zeatin, and several riboside conjugates that are generally classified as storage forms of the phytohormone (Hirose et al., 2007). *Trans*-zeatin, the main active CK generated via the methylerythritol phosphate pathway, was below detection limits in our leaf samples.

No major differences were observed in the levels of *cis*-zeatin, its riboside and *N*6-isopentenyladenosine riboside between leaf 1 and leaf 7 of WT plants (Supplementary Figure S10). Contents of these three CKs were higher than WT in *pfld* leaves but not in their *cfld*1-4 siblings. Interestingly, the main storage form, dihydrozeatin riboside, which represents more than 80% of all riboside conjugates, showed a major increase in WT leaf 7 compared to leaf 1, with this effect being significantly impaired in *pfld* lines (Supplementary Figure S10 and Supplementary Table S5).

Auxins are also reported to decline in senescing leaves (Kim et al., 2011). Contents of IAA, the free active form of the hormone, were similar in leaf 1 of all lines and lower in leaf 7 (Supplementary Figure S11). This decline was partially prevented in *pfld* leaves, although the protection was moderate and not statistically significant. The IAA precursor indole-3-acetamide and two of the main inactive auxins, 2-oxoindole-3-acetic acid (2-oxoIAA) and indole-3-acetyl-L-alanine, also declined with age (Supplementary Figure S11). Interestingly, leaf 1 of *pfld* plants showed higher contents of indole-3-acetamide and lower levels of 2-oxoIAA (Supplementary Figure S11 and Supplementary Table S5).

### Senescence Marker Genes Exhibited Delayed Expression in *pfld* Plants

**FIGURE 9:**
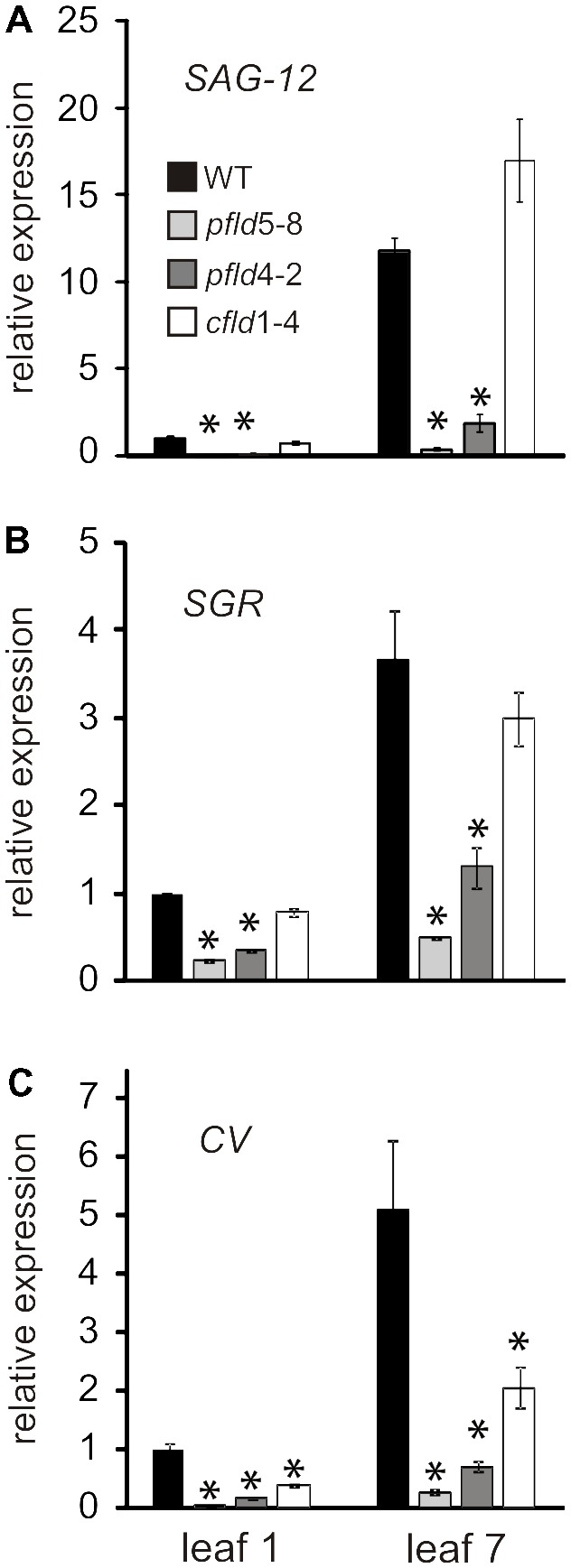
Fld expression in plastids delayed induction of senescence-associated marker genes during leaf senescence. Transcript levels of *Senescence-Associated Gene 12* (*SAG12*, **A**), *Stay-green* (*SGR*, **B**) and *Chloroplast Vesiculation* (*CV*, **C**) in leaves 1 and 7 of WT, *pfld* and *cfld* plants at 73 dpg were determined by qRT-PCR. Values are means ± SE of fold-changes relative to the levels of leaf 1 from WT plants (*n* = 6). Asterisks indicate statistically significant differences with respect to the wild type (ANOVA, *P* < 0.05).

As a further indicator of leaf senescence, we measured the transcript abundance of known marker genes: *SAG12, CV* and *SGR* by qRT-PCR. The product of *SAG12* is a vacuolar cysteine protease involved in protein turnover and mobilization (Carrión et al., 2013), whereas the *SGR* protein is a Mg-dechelatase catalyzing a key step of *Chl* catabolism in chloroplasts (Shimoda et al., 2016). The *CV* gene encodes a protein involved in plastid degradation during senescence and abiotic stress (Wang and Blumwald, 2014). Transcripts of the three genes were reported to increase during senescence (Carrión et al., 2013; Wang and Blumwald, 2014). In agreement with those observations, they exhibited higher levels in leaf 7 of WT and *cfld*1-4 plants relative to leaf 1, whereas Fld presence in chloroplasts of *pfld* plants largely prevented this induction (**Figures 9A–C**). The results indicate that the whole mechanism of leaf senescence is delayed in *pfld* plants.

## Discussion

Fld expression in chloroplasts of transgenic tobacco plants significantly delayed leaf senescence, as indicated by lower decay of *Chl, Car* and proteins (**Figures 1**, **2**, **5**), and differential preservation of cell structure and viability (**Figure 4** and Supplementary Figures S6, S7). The gradual loss of *Chl* during leaf senescence is normally accompanied by a decrease in photosynthetic activity along with the degradation of structural components of the antennae and reaction centers in the thylakoid membranes (Guiamet et al., 2002). In the case of *pfld* leaves, protection of pigments and proteins also led to maintenance of intact antennae, reaction centers and photosynthetic activities for extended time periods (**Figures 6**, **7**). Plastid-targeted Fld also protected tobacco leaf disks against SA-induced senescence (Supplementary Figure S4).

Metabolite profiling was carried out to determine the impact of chloroplast Fld on central metabolic routes during senescence. In general, the presence of the flavoprotein had a more significant effect on nitrogen than on carbon metabolism (**Figure 8** and Supplementary Tables S3, S4). The age-dependent decline of Suc was alleviated by Fld presence (**Figure 8** and Supplementary Table S3), suggesting that *pfld* transformants retained a better capacity for sugar export to sinks elsewhere in the plant. While the effect of Fld on sugar phosphates and intermediates of the TCA cycle was less significant, the overall results showed that the profiles of metabolites related to major carbon pathways obtained in *pfld* plants resembled those of younger leaves in the wild type (**Figure 8** and Supplementary Table S3), concurring with the general delay of senescence caused by Fld presence. Interestingly, malate increased in leaf 7 of all lines, but accumulated at significantly higher levels in *pfld* plants relative to WT and *cfld*1-4 siblings. An efficient recycling of carbon and nitrogen across cellular compartments depends in part on a concerted action of the chloroplastic and mitochondrial malate/oxaloacetate redox shuttles (Foyer et al., 2011). Higher malate content in aging *pfld* leaves is in agreement with increased dissipation of the excess of reducing power via the chloroplast malate valve. In fact, we have demonstrated that plastid-targeted Fld is able to sustain high malate dehydrogenase activity under abiotic stress conditions by interacting with the Trx/Fd-Trx reductase system (Tognetti et al., 2006).

Most amino acids showed lower levels in WT leaf 7 compared to leaf 1 (**Figure 8** and Supplementary Table S4), in good agreement with the observations reported by Li et al. (2016) for Chinese tobacco cultivars. This decline was largely prevented by Fld presence in chloroplasts (**Figure 8** and Supplementary Table S4). Ammonium incorporation into amino acids is mediated by glutamine synthetases, with isoenzymes present in chloroplasts (GS2) and cytosol (GS1). GS1 uses ammonium derived from primary nitrogen uptake and internal recycling pathways, whereas GS2 is involved in photosynthetic and photorespiratory nitrogen metabolism (Bernard and Habash, 2009). Masclaux et al. (2000) reported that GS2-encoding mRNA and GS2 protein levels declined steadily during senescence of tobacco leaves. Transcripts encoding GS1 also decreased in the course of leaf development until the early phase of senescence, to significantly increase thereafter (Masclaux et al., 2000; Uzelac et al., 2016). As indicated, leaf 7 of WT and *cfld*1-4 plants was in the initial stages of senescence at 73 dpg, and accordingly, we observed low levels of both GS1 and GS2 compared to leaf 1 (**Figure 5C**). Partial protection conferred by plastid-targeted Fld to this decline could explain the differential preservation of amino acids in aging *pfld* leaves.

The Gln/Glu, Asn/Asp and Gly/Ser relationships have been customarily related to nitrogen mobilization and photorespiratory activity, respectively (Wingler et al., 2000; Havé et al., 2016). Both processes were reported to increase during senescence (Havé et al., 2016; Li et al., 2016). Accordingly, we observed significantly higher ratios in leaf 7 of WT plants compared to leaf 1 (Supplementary Figure S9). The increase in the Gln/Glu and Asn/Asp ratios was partially prevented by Fld expression in chloroplasts (Supplementary Figures S9A,B), indicating lower nitrogen turnover in the *SGR* leaves of the transformants, and consistent with differential preservation of amino acids (**Figure 8**) and proteins (**Figure 5**) in these plants. The age-dependent increase of the Gly/Ser relationship was also down-regulated by plastid-targeted Fld, suggesting lower induction of photorespiration in leaf 7 of *pfld* plants (Supplementary Figure S9C).

Molecular markers such as *SAG12, CV* and *SGR* exhibited expression patterns that paralleled the delayed senescence phenotype of *pfld* plants (**Figure 9**). This result, together with differential preservation of cellular viability and metabolic activities indicates that the overall process of senescence was retarded in *pfld* plants. *SGR* mutants have been described in many species (Gregersen et al., 2013), and were categorized in two major groups: functional and non-functional, also termed cosmetic (Thomas and Howarth, 2000). The difference between these categories is whether retention of greenness is coupled to preservation of metabolic capacity. Cosmetic *SGR* mutants show normal senescence behavior but retain green color, indicating that they are just defective in *Chl* breakdown. Since *pfld* plants showed a significant delay of all structural, functional and molecular symptoms of senescence, they conform to the functional *SGR* category. This effect of plastid-targeted Fld on senescence is independent of the higher *Chl* and *Car* levels per leaf cross-section reported for these transformants (Tognetti et al., 2006; Ceccoli et al., 2012; **Figure 2**). Increased cell densities in *pfld* leaves most likely explain their pigmentation phenotype (Supplementary Figure S2). Research is currently underway to determine the mechanisms by which Fld presence in chloroplasts affects leaf cell size and packing.

Besides extended preservation of structural integrity and biochemical functions, aging *pfld* leaves showed a remarkable decrease of chloroplast ROS accumulation (**Figure 3** and Supplementary Figure S5), suggesting that these reactive species could be involved in modulating the progress of leaf senescence. The molecular mechanisms of senescence-associated cell demise have been thoroughly studied in animal systems, where mitochondrial ROS make a key contribution. Release of cytochrome *c* from the inner mitochondrial membrane of death-targeted cells results in over-reduction of up-chain respiratory transporters and increased ROS propagation by adventitious electron transfer to O_2_ (Fuchs and Steller, 2011). Mitochondria also participate in triggering age-associated cell death in plants, especially in non-photosynthetic organs such as petals (Rhoads et al., 2006; Rogers and Munné-Bosch, 2016; Muñoz and Munné-Bosch, 2018), but chloroplasts are the main source of ROS in illuminated leaves and accordingly, the contribution of chloroplast-generated ROS to leaf senescence seemed reasonable (Ambastha et al., 2015; Van Aken and Van Breusegem, 2015; Muñoz and Munné-Bosch, 2018).

There are many reports supporting the participation of ROS in the execution of the senescence program, without discrimination of their origin (Van Breusegem and Dat, 2006; Zentgraf, 2007; Niewiadomska et al., 2009; Sedigheh et al., 2011; Jajic et al., 2015). ROS may coordinate senescence progress via chloroplast degradation through the activity of ATAF1, a H_2_O_2_-responsive transcriptional regulator that induces expression of the senescence-promoting transcription factor ORESARA1 while repressing the *GLK1* gene involved in photosynthesis and chloroplast maintenance (Garapati et al., 2015).

The contribution of chloroplast ROS to cell death is documented for various stress conditions (Samuilov et al., 2003; Zurbriggen et al., 2009; Rossi et al., 2017). Instead, empirical evidence supporting a direct link between leaf senescence and chloroplast redox chemistry is scant. Zapata et al. (2005) reported that tobacco *ndhF* mutants deficient in chloroplast NAD(P)H dehydrogenase activity exhibited delayed senescence (Zapata et al., 2005). The dehydrogenase was assumed to deliver extra reducing equivalents into the ETC, which could be misrouted to oxygen in aging leaves. Later research, however, showed that Arabidopsis mutants with impaired NAD(P)H dehydrogenase activity failed to show a senescence phenotype (Wang et al., 2015), suggesting that the effect observed with the *ndhF* plants was likely related to the particular subunit or species assayed, and challenging the proposed mechanism. Decrease in tocopherol levels by silencing the rate-limiting enzyme homogentisate phytyltransferase led to higher ROS levels and accelerated senescence in tobacco (Abbasi et al., 2009). However, the cellular location of the augmented ROS was not determined and the differential effect displayed by the transgenic plants was only evident after flowering (Abbasi et al., 2009). A possible involvement of chloroplast ROS on senescence was suggested by the phenotype of wheat lines expressing a chloroplast protein kinase that inactivates thylakoid-bound ascorbate peroxidase and exhibited anticipated leaf senescence (Gou et al., 2015). ROS levels were increased in these plants, but once again their cellular compartmentation was not determined (Gou et al., 2015). Our results, then, represent the first report showing a strong correlation between chloroplast-generated ROS (**Figure 3**) and natural leaf senescence (**Figures 1**, **2**), providing compelling evidence to the notion that chloroplast redox chemistry affects the senescence program at an early stage and at a hierarchically high level of developmental decisions.

Reactive oxygen species can be generated in various chloroplast reactions, most conspicuously as byproducts of photosynthetic electron transport. Electron and energy transfer to oxygen occur even under optimal photosynthetic conditions, but ROS build-up is limited by the action of several antioxidant systems present in the chloroplast, involving both scavenging and dissipative mechanisms. However, if proper distribution of reducing equivalents throughout the ETC is perturbed by biotic or abiotic stresses or in response to developmental programs such as senescence, the rate of leakage increases dramatically, overcoming the control devices and leading to ROS propagation (Mignolet-Spruyt et al., 2016; Sewelam et al., 2016). It is worth noting, in this context, that Fd levels decreased with age in WT leaves (**Figure 5C**), concurring with earlier observations in tomato (John et al., 1997). As indicated, Fd is the terminal protein acceptor of the ETC and the functional counterpart of Fld in plants (Pierella Karlusich et al., 2014). Fd down-regulation would cause acceptor side limitation, excess excitation on the ETC and over-reduction of chain intermediates. Probability of energy and electron transfer to oxygen would increase, with concomitant ROS propagation which might provide signals for senescence progression. In this sense, Fd decline could play a role analogous to that of cytochrome *c* release during animal senescence (Fuchs and Steller, 2011).

Fld can limit chloroplast ROS build-up through three major mechanisms: (i) by providing an alternative, productive electron sink to declining Fd levels drives away part of the reducing equivalents from oxygen and relieves the excess of excitation energy over the ETC, (ii) boosting endogenous antioxidant systems (Tognetti et al., 2006), and (iii) keeping chemical scavengers in a more reduced state (Zurbriggen et al., 2009; Li et al., 2017). Since Fld limits ROS generation rather than scavenging them once formed, all photosynthesis-associated ROS are down-regulated (Pierella Karlusich et al., 2014).

Reactive oxygen species and phytohormones are major mediators of physiological responses throughout the plant lifespan, and their intra- and intercellular spatial and temporal distribution among different tissues and organs enables adequate responses to distinct developmental and environmental requirements (Tognetti et al., 2012). It is generally accepted that CK levels decline during leaf senescence to signal the remobilization of nutrients from vegetative tissues to reproductive organs (Talla et al., 2016). Besides degradation, CKs can be deactivated by *N*-glycosylation, and several reports have shown that the levels of *N*-glucosides increase in senescing tissues (Šmehilová et al., 2016; Uzelac et al., 2016). The age-response of active and storage CK forms (free species and ribosides) is instead complex and does not appear to follow a simple correlation with the progress of senescence. In Arabidopsis leaves, total active CKs did not change with age, even at advanced senescence stages (Šmehilová et al., 2016). Moreover, CK profiling studies in free-growing aspen trees (*Populus tremula*) over three consecutive years showed that the total pool of CKs belonging to the *cis*-zeatin, *trans*-zeatin and dihydrozeatin groups actually increased during the initiation and progression of autumn senescence. A similar lack of correlation between CK accumulation and timing of senescence has been reported for lettuce (*Lactuca sativa L.*) by McCabe et al. (2001). These evidences challenge the notion that the onset of senescence is triggered by a decline in CK levels (Edlund et al., 2017). In line with those reports, we did not observe a decrease of the bioactive CK *cis*-zeatin in WT leaf 7 relative to leaf 1 (Supplementary Figure S10). Our results also agree with other CK determinations in senescing tobacco leaves which failed to show age-dependent changes in several active forms including *cis*-zeatin (Uzelac et al., 2016). Noteworthy, plants accumulating Fld in chloroplasts contained higher levels of *cis*-zeatin in both leaf 1 and 7 (Supplementary Figure S10), but the contribution of this accumulation to the *SGR* phenotype of the transformants remains to be determined.

Levels of active auxins are also finely controlled by conjugation/deconjugation and degradation (Tognetti et al., 2012). Auxins were down-regulated with age in WT tobacco plants, but the effect of plastid-targeted Fld on this process was modest (Supplementary Figure S11). Kim et al. (2011) have suggested that changes in auxin gradients rather than the auxin concentration itself may modulate senescence development. Although Fld expression in chloroplasts had little consequences for total auxin levels, it did affect auxin homeostasis as evidenced by the higher contents of IAM and lower levels of 2-oxoIAA in *pfld* leaves (Supplementary Figure S11 and Supplementary Table S5).

While elucidation of the reciprocal interactions between chloroplast-located ROS and phytohormones requires more extensive research, we propose that the increased metabolic activity, chlorophyll retention and lower ROS production of *pfld* chloroplasts could sustain higher CK and auxin pools, delaying the age-dependent changes in the levels of these phytohormones (Kim et al., 2011).

In addition to its basic interest as a fundamental stage of plant development, the timing and progression of senescence have profound agronomical consequences. Recognition of chloroplast ROS and/or redox status as a factor that might influence the onset and progress of senescence provides novel tools to investigate and manipulate the molecular mechanisms underlying this key developmental pathway with significant implications in biology and agronomy.

## Author Contributions

AL, VT, MH, and NC conceived the original research plans. MLM, AL, JG, VT, MM, MH, and NC designed the experiments. MLM, AL, JG, MM, and MH performed the experiments. MLM, AL, JG, VT, MM, MH, and NC analyzed the data. MLM, AL, JG, VT, MM, MH, and NC wrote the manuscript.

## Conflict of Interest Statement

The authors declare that the research was conducted in the absence of any commercial or financial relationships that could be construed as a potential conflict of interest.

## Footnotes

1 http://www.matrixscience.com

2 www.bioinformatics.nl/primer3plus/

## Abbreviations

*Car*: carotenoids
*Chl*: chlorophyll
CK: cytokinin
*CV*: *Chloroplast Vesiculation*
DCFDA: 2′,7′-dichlorodihydrofluorescein diacetate
Fd: ferredoxin
Fld: flavodoxin
Fru: fructose
FW: fresh weight
Glc: glucose
IAA: indole-3-acetic acid
PEP: phospho-*enol*-pyruvate
qRT-PCR: quantitative reverse-transcription PCR
ROS: reactive oxygen species
SAG: senescence-associated gene
*SGR*: *Stay-Green*
Suc: sucrose
Trx: thioredoxin
TTC: 2,3,5-triphenyltetrazolium chloride
UPLC: ultra-pressure liquid chromatography
WT: wild-type
