# Supplementary material for: Expression of a Plastid-Targeted Flavodoxin Decreases Chloroplast Reactive Oxygen Species Accumulation and Delays Senescence in Aging Tobacco Leaves
Source: Front Plant Sci. 2018 Jul 17;9:1039. doi: 10.3389/fpls.2018.01039 (PMC6056745; doi:10.3389/fpls.2018.01039)
Supplement: Supplementary file 1 [file Image_1.PDF]

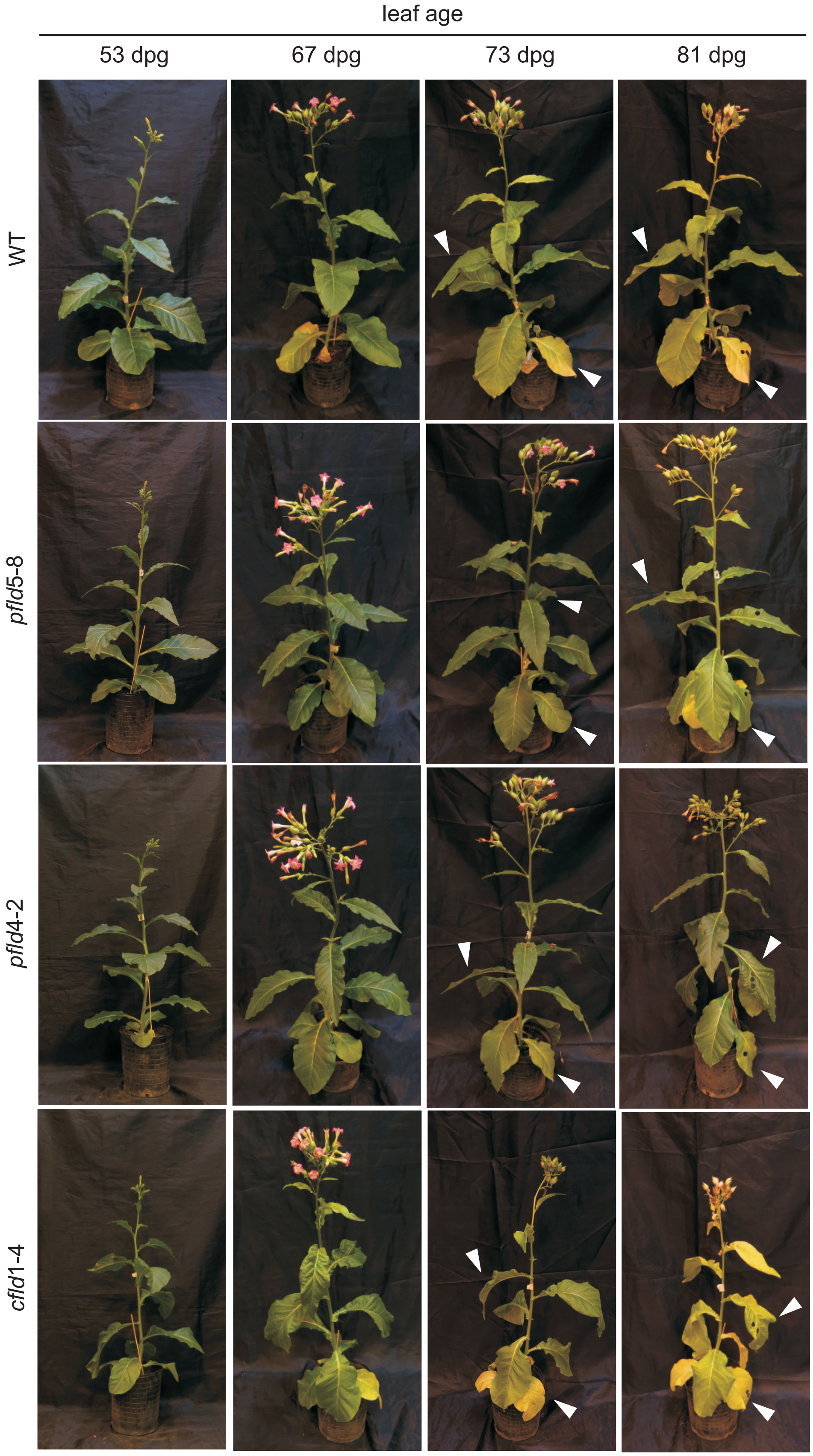

Supplementary Figure S1. Phenotypes of WT, *pfld* and *cfld* plants at different dpg. White arrowheads indicate leaves 1 and 7.
