# Supplementary material for: Expression of a Plastid-Targeted Flavodoxin Decreases Chloroplast Reactive Oxygen Species Accumulation and Delays Senescence in Aging Tobacco Leaves
Source: Front Plant Sci. 2018 Jul 17;9:1039. doi: 10.3389/fpls.2018.01039 (PMC6056745; doi:10.3389/fpls.2018.01039)
Supplement: Supplementary file 2 [file Image_2.PDF]

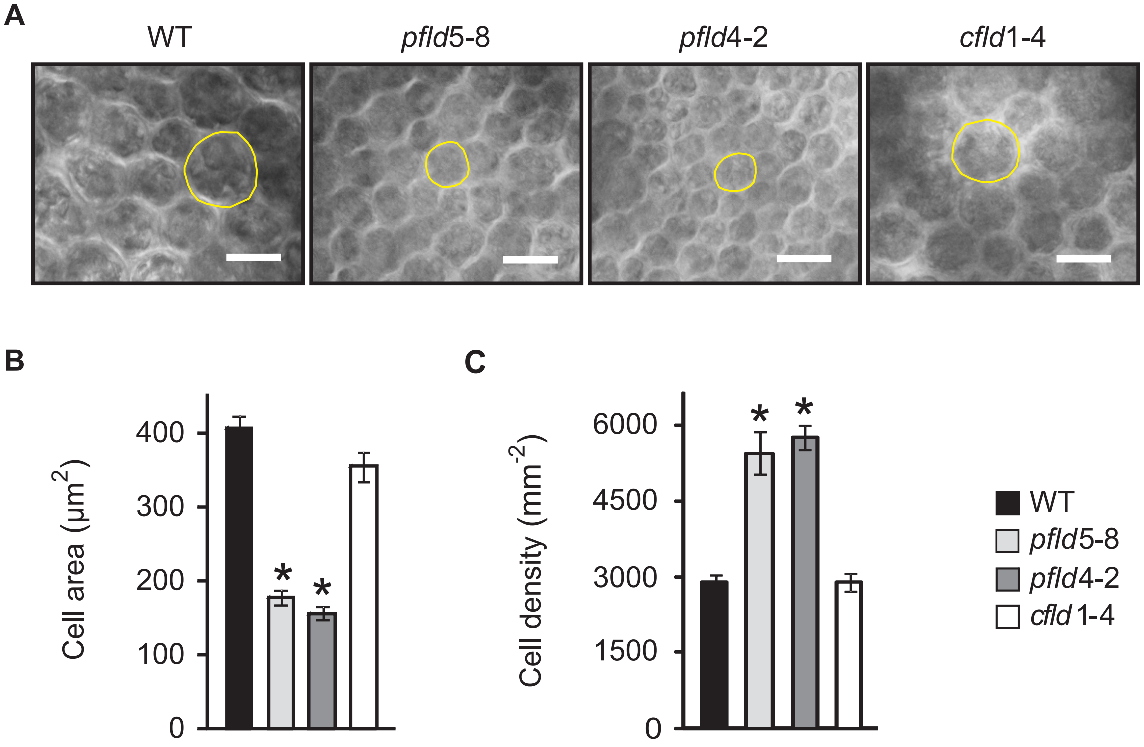

Supplementary Figure S2. Fld expression in chloroplasts affected cell size and organization in leaves of *pfld* plants. Size (A,B) and number per mm^2 (C) of mesophyll cells were estimated in leaf 1 of WT, *pfld* and *cfld*1-4 plants at 73 dpg. Bar = 20 µm. Sample preparation is described in Materials and Methods. Data shown are means ± SE (n = 7-9). Asterisks indicate significant differences with respect to the wild type (ANOVA, P < 0.01).
