# Supplementary material for: Expression of a Plastid-Targeted Flavodoxin Decreases Chloroplast Reactive Oxygen Species Accumulation and Delays Senescence in Aging Tobacco Leaves
Source: Front Plant Sci. 2018 Jul 17;9:1039. doi: 10.3389/fpls.2018.01039 (PMC6056745; doi:10.3389/fpls.2018.01039)
Supplement: Supplementary file 3 [file Image_3.PDF]

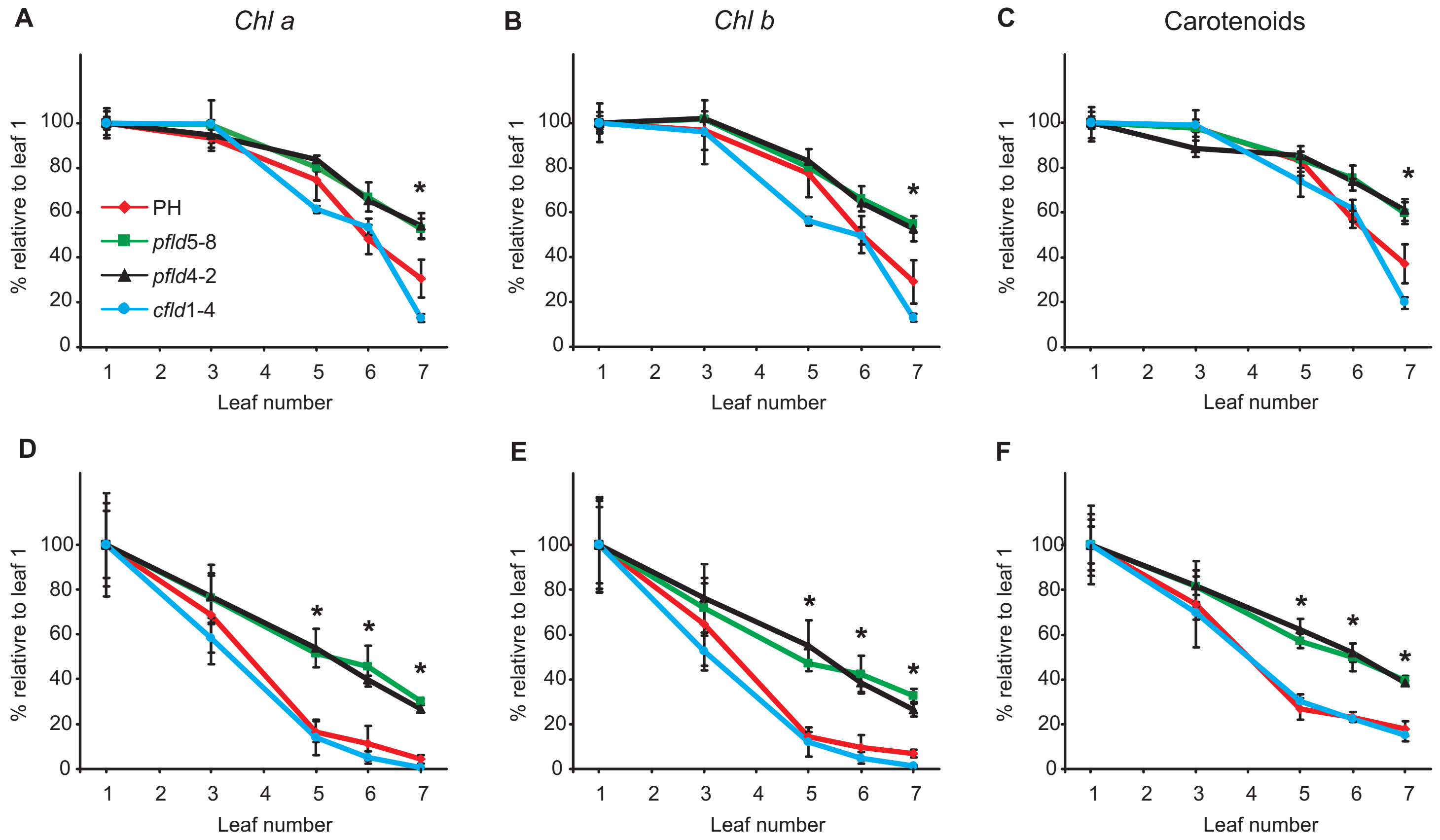

Supplementary Figure S3. Fld expression in plastids delayed degradation of *Chl a* (A, D), *Chl b* (B, E) and carotenoids (C, F) during leaf senescence. Plants were assayed at 73 and 81 dpg. Pigment levels are expressed as the percentage of the corresponding values determined in leaf 1 of the same plant. Data shown are means ± SE (n = 3). Asterisks indicate significant differences (ANOVA, $P < 0.05$).
