# Supplementary material for: Expression of a Plastid-Targeted Flavodoxin Decreases Chloroplast Reactive Oxygen Species Accumulation and Delays Senescence in Aging Tobacco Leaves
Source: Front Plant Sci. 2018 Jul 17;9:1039. doi: 10.3389/fpls.2018.01039 (PMC6056745; doi:10.3389/fpls.2018.01039)
Supplement: Supplementary file 4 [file Image_4.PDF]

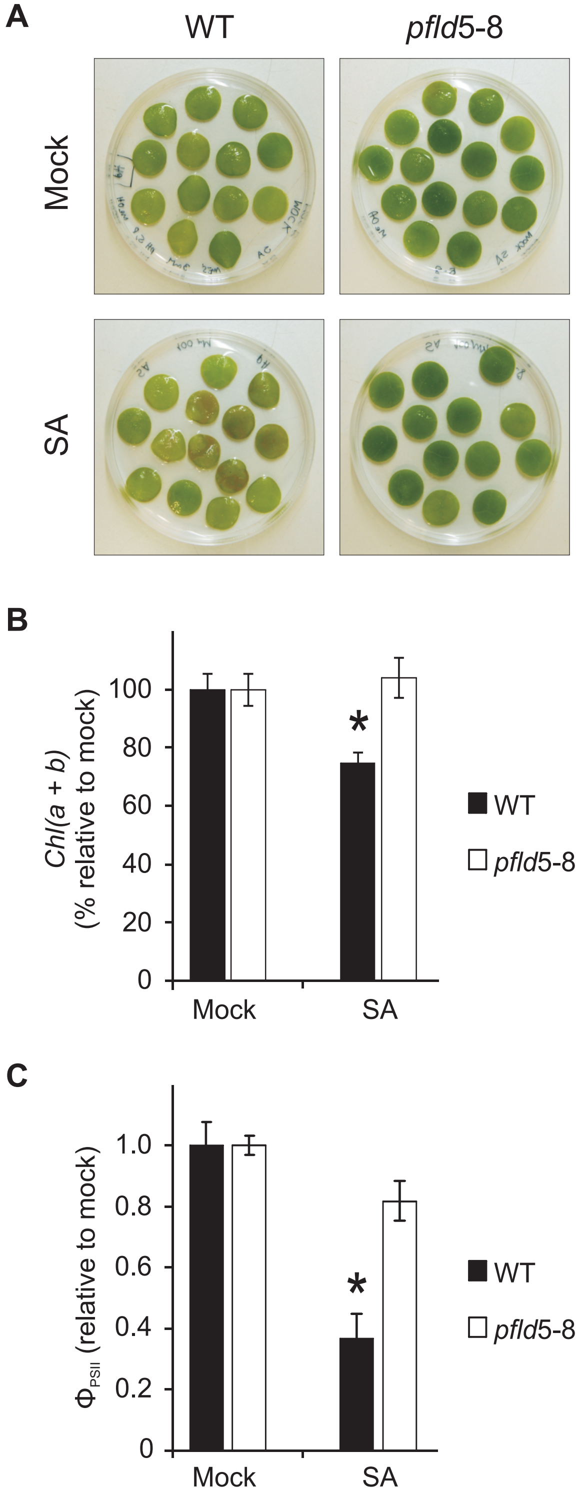

Supplementary Figure S4. Fld expression in chloroplasts delays SA-induced leaf senescence. Discs (1 cm in diameter) were punched from leaf 1 of 12-15 independent plants at 63 dpg, floated in 3 mM MES pH 5.8 containing 100 µM SA or an equal volume of buffer (Mock), and incubated under growth chamber conditions (see Materials and Methods). Pictures were taken after 6 days (A), and measurements of total chlorophyll (*Chl(a + b)*) contents (B) and the quantum yield of photosystem II (Φ_{PSII}) (C) were carried out at that time. Values presented are means ± SE relative to the "Mock" condition. Asterisks indicate significant differences (ANOVA, $P < 0.05$).
