# Supplementary material for: Expression of a Plastid-Targeted Flavodoxin Decreases Chloroplast Reactive Oxygen Species Accumulation and Delays Senescence in Aging Tobacco Leaves
Source: Front Plant Sci. 2018 Jul 17;9:1039. doi: 10.3389/fpls.2018.01039 (PMC6056745; doi:10.3389/fpls.2018.01039)
Supplement: Supplementary file 5 [file Image_5.PDF]

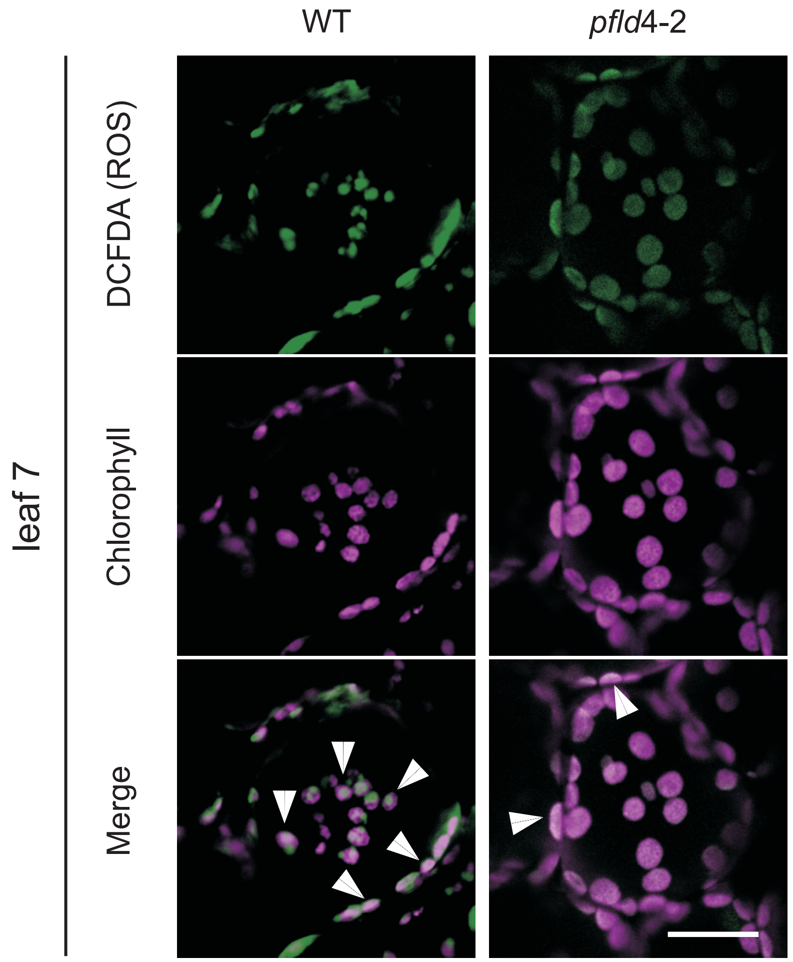

Supplementary Figure S5. Expression of a plastid-targeted Fld in transgenic tobacco suppressed ROS build-up in chloroplasts of ageing leaves. Magnification of leaf 7 tissue stained with the ROS-sensitive probe DCFDA to show a single cell of WT and *pfld*4-2 genotypes. Bar = 20 µm. ROS (green), chlorophyll (magenta) and merge images are shown. Arrowheads show merge of *Chl* and ROS-derived signals in chloroplasts. All other conditions are those indicated in the legend to Figure 3.
