# Supplementary material for: Expression of a Plastid-Targeted Flavodoxin Decreases Chloroplast Reactive Oxygen Species Accumulation and Delays Senescence in Aging Tobacco Leaves
Source: Front Plant Sci. 2018 Jul 17;9:1039. doi: 10.3389/fpls.2018.01039 (PMC6056745; doi:10.3389/fpls.2018.01039)
Supplement: Supplementary file 6 [file Image_6.PDF]

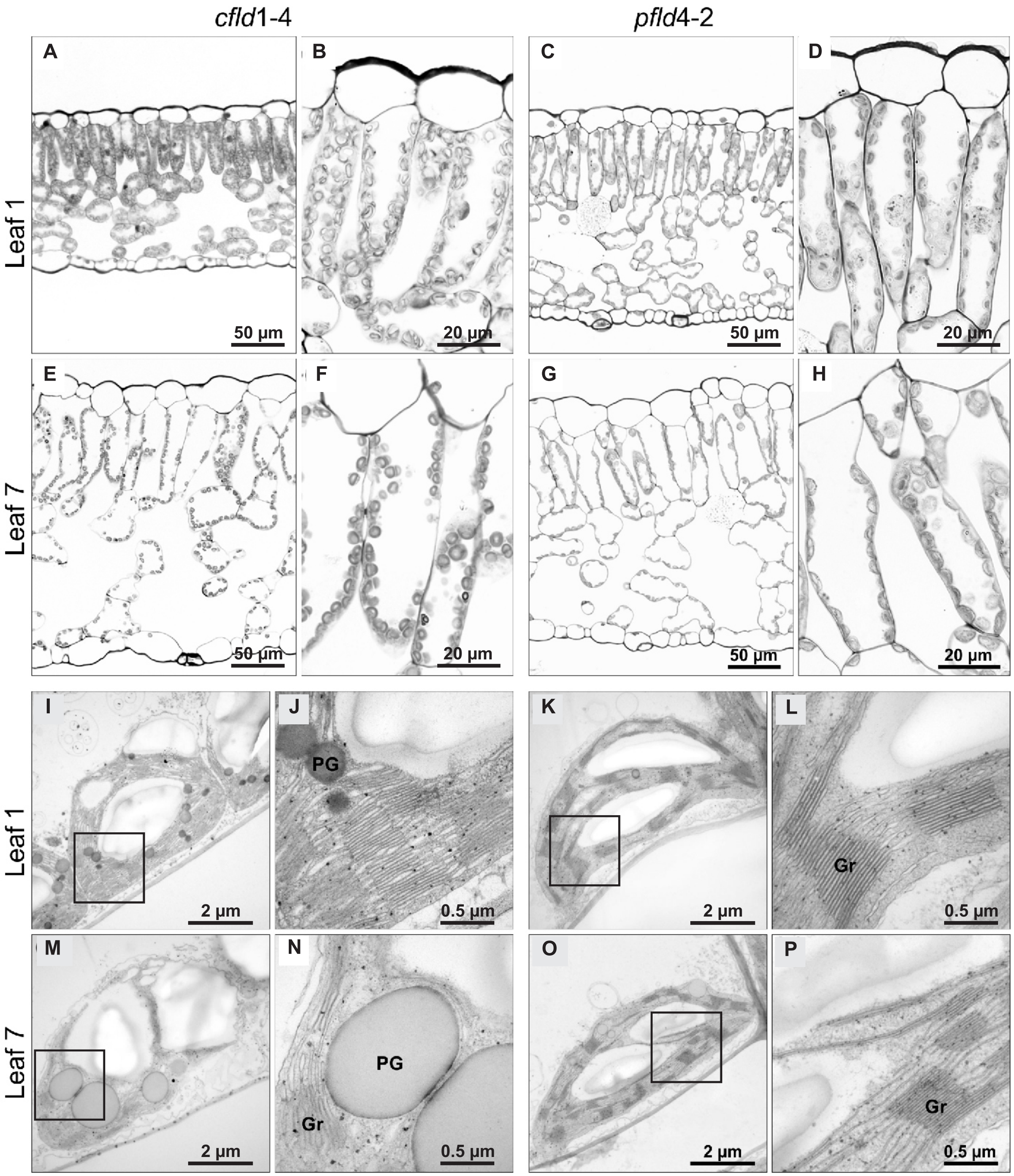

Supplementary Figure S6. Fld expression in plastids prevented loss of tissue and chloroplast integrity during leaf senescence. Structural analysis of leaf cells and chloroplasts in cross-sections of leaf 1 and 7 from cfld1-4 and pfld4-2 lines at 73 dpg. Light microscopic images (A-H), and TEM micrographs (I-P) of palisade parenchyma tissue showing cells and chloroplasts at two different magnifications. Gr = grana, PG = plastoglobuli.
