# Supplementary material for: Expression of a Plastid-Targeted Flavodoxin Decreases Chloroplast Reactive Oxygen Species Accumulation and Delays Senescence in Aging Tobacco Leaves
Source: Front Plant Sci. 2018 Jul 17;9:1039. doi: 10.3389/fpls.2018.01039 (PMC6056745; doi:10.3389/fpls.2018.01039)
Supplement: Supplementary file 7 [file Image_7.PDF]

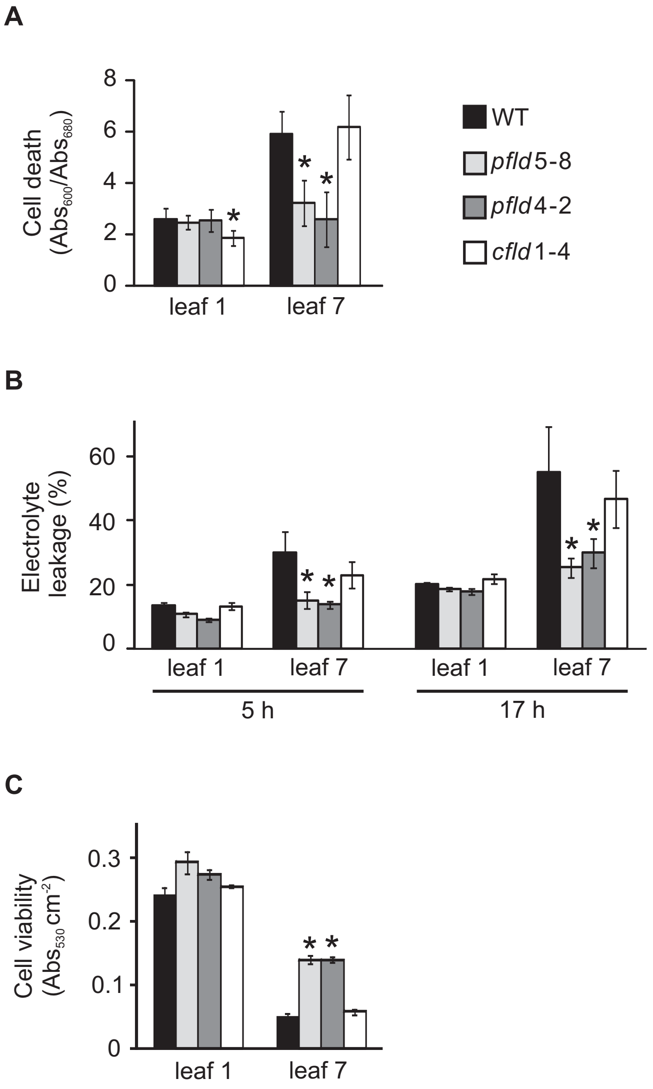

Supplementary Figure S7. Plastid-targeted Fld reduced cell death and viability loss during leaf senescence. (A) Cell death estimated by Evans Blue staining. (B) Electrolyte leakage in leaf discs measured after 5 h and 17 h of incubation in distilled water. (C) Cell viability determined by the reduction of TTC in leaf discs. Details on the methods used are given in Materials and Methods. Data shown are means ± SE of 4 biological replicates. Asterisks indicate statistically significant differences with respect to WT leaves (ANOVA, $P < 0.05$). Plants were assayed at 73 dpg.
