# Supplementary material for: Expression of a Plastid-Targeted Flavodoxin Decreases Chloroplast Reactive Oxygen Species Accumulation and Delays Senescence in Aging Tobacco Leaves
Source: Front Plant Sci. 2018 Jul 17;9:1039. doi: 10.3389/fpls.2018.01039 (PMC6056745; doi:10.3389/fpls.2018.01039)
Supplement: Supplementary file 8 [file Image_8.PDF]

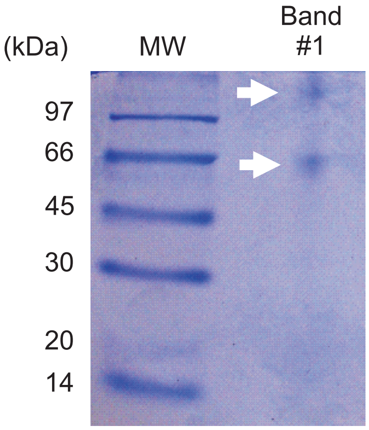

Supplementary Figure S8. Major protein components of band #1 from the green gel depicted in Figure 6. Band #1 was excised, and proteins were resolved by SDS-PAGE and stained with Coomassie Brilliant Blue. Arrows indicate the major bands. MW = molecular weight standards.
