# Supplementary material for: Expression of a Plastid-Targeted Flavodoxin Decreases Chloroplast Reactive Oxygen Species Accumulation and Delays Senescence in Aging Tobacco Leaves
Source: Front Plant Sci. 2018 Jul 17;9:1039. doi: 10.3389/fpls.2018.01039 (PMC6056745; doi:10.3389/fpls.2018.01039)
Supplement: Supplementary file 9 [file Image_9.PDF]

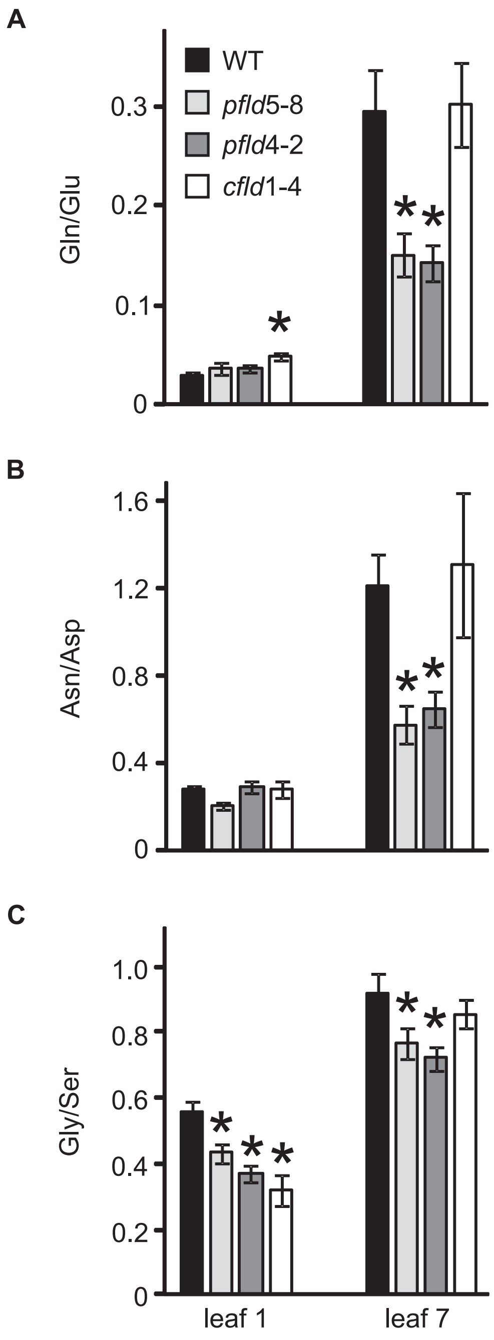

Supplementary Figure S9. Fld expression in chloroplasts differentially affected the levels of amino acids related to N mobilization and photorespiration in ageing tobacco leaves. Ratios of Gln to Glu (A), Asn to Asp (B) and Gly to Ser (C) were calculated for leaves 1 and 7 of WT, *pfld* and *cfld* plants. Details on amino acid determinations in leaf extracts are given in Materials and Methods. Data reported are means ± SE (n = 8-10). Asterisks indicate values significantly different from the corresponding leaf of WT plants ($P < 0.05$) using ANOVA.
