# Supplementary material for: Expression of a Plastid-Targeted Flavodoxin Decreases Chloroplast Reactive Oxygen Species Accumulation and Delays Senescence in Aging Tobacco Leaves
Source: Front Plant Sci. 2018 Jul 17;9:1039. doi: 10.3389/fpls.2018.01039 (PMC6056745; doi:10.3389/fpls.2018.01039)
Supplement: Supplementary file 10 [file Image_10.PDF]

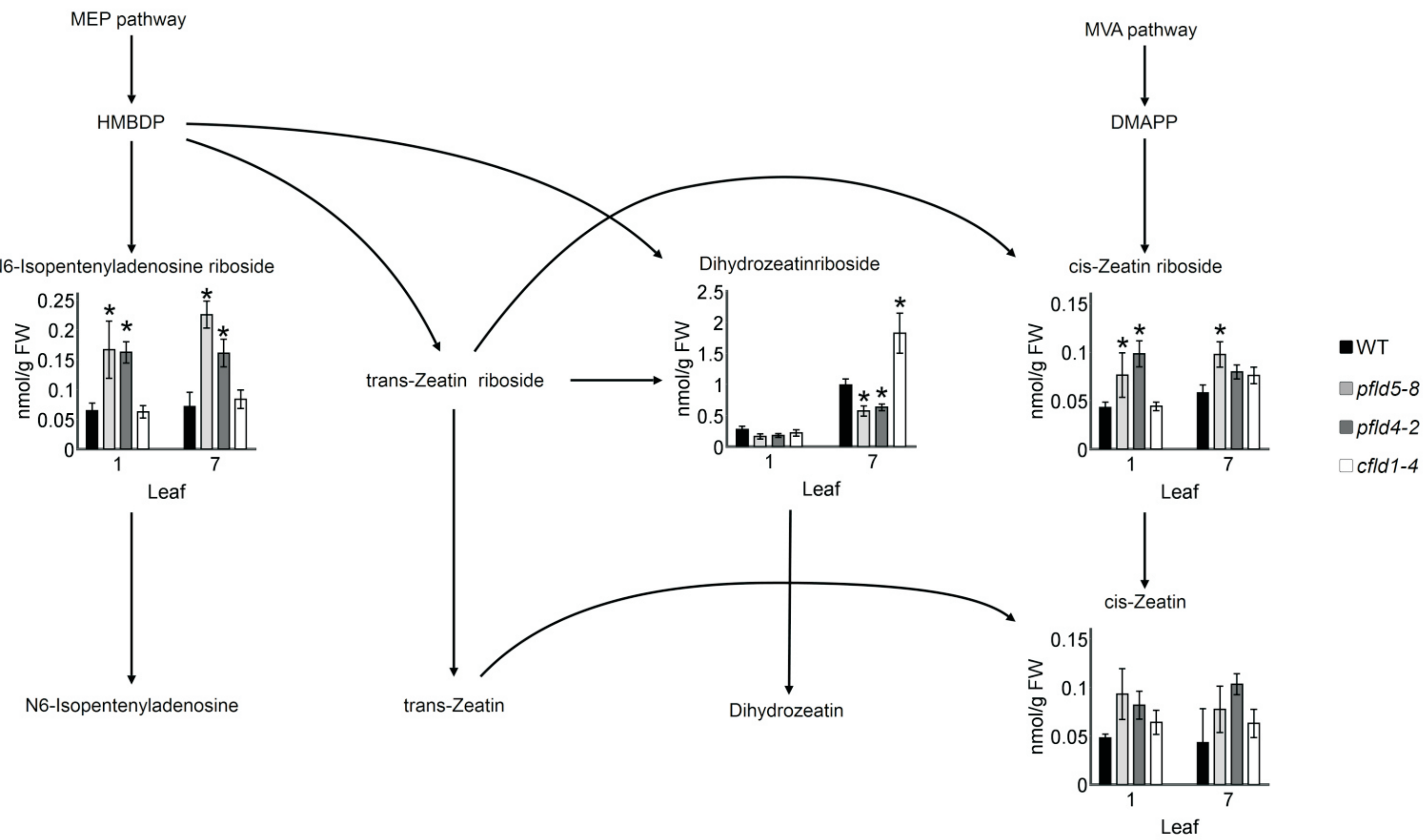

Supplementary Figure S10. Plants expressing plastid-targeted Fld contained higher levels of active cytokinins. CKs were measured in leaf tissue from WT, *pfld* and *cfld* plants at 73 dpg. Values shown are means ± SE (n = 3-5). Asterisks indicate significant differences with respect to the wild type (ANOVA, $P < 0.05$). The graph was created using the visualization system VANTED (Junker et al., 2006). MVA, mevalonate; MEP, methylerythritol-4-phosphate; DMAPP, dimethylallyl pyrophosphate; HMBDP, (E)-4-hydroxy-3-methyl-but-2-enyl diphosphate. Units in the ordinates are nmol g^{-1} FW.
