# Supplementary material for: Expression of a Plastid-Targeted Flavodoxin Decreases Chloroplast Reactive Oxygen Species Accumulation and Delays Senescence in Aging Tobacco Leaves
Source: Front Plant Sci. 2018 Jul 17;9:1039. doi: 10.3389/fpls.2018.01039 (PMC6056745; doi:10.3389/fpls.2018.01039)
Supplement: Supplementary file 11 [file Image_11.PDF]

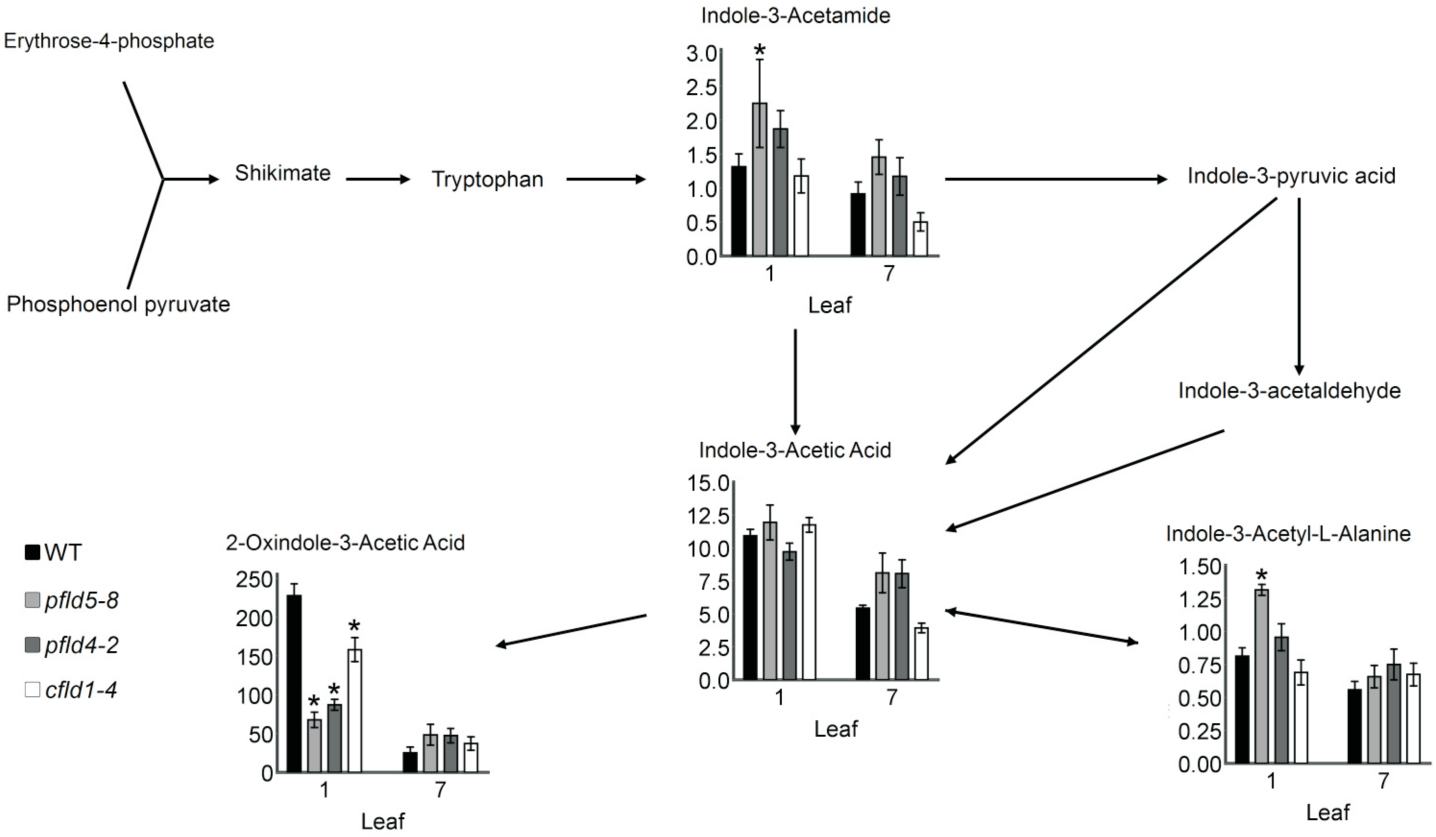

Supplementary Figure S11. Plastid-targeted Fld partially prevented age-dependent decline of endogenous auxin levels in leaves of pfld plants. Auxins were measured in leaf tissue from WT, *pfld* and *cfld* plants at 73 dpg. Values shown are means ± SE (n = 3-5). Asterisks indicate significant differences with respect to the wild type (ANOVA, $P < 0.05$). The graph was created using the visualization system VANTED (Junker et al., 2006). Units in the ordinates are nmol g^{-1} FW.
