# Supplementary material for: Expression of a Plastid-Targeted Flavodoxin Decreases Chloroplast Reactive Oxygen Species Accumulation and Delays Senescence in Aging Tobacco Leaves
Source: Front Plant Sci. 2018 Jul 17;9:1039. doi: 10.3389/fpls.2018.01039 (PMC6056745; doi:10.3389/fpls.2018.01039)
Supplement: Supplementary file 12 [file Table_1.PDF]

Supplementary Table S1. Phenotypic analysis of WT, *pfld* and *cfld* plants. Plants were cultivated in soil under growth chamber conditions as described in Materials and Methods. Pigment levels were determined at 73 and 81 dpg. Values are presented as means ± SE (n = 4-6). Leaves and fruits used for FW and DW determinations were collected at 83-85 dpg. Values given are means ± SE of 10 individual plants of each of the four lines. At least 50 fruits per line were used for weight measurements. Significant differences (ANOVA, P < 0.05) between transgenic and WT plants are shown in bold.

Phenotypic character			WT	*pfld* 5-8	*pfld* 4-2	*cfld* 1-4
**Pigment levels (mg m^{-2})**						
73 dpg	leaf 1	*Chl a*	205 ± 11	**263 ± 13**	**301 ± 11**	226 ± 18
		Chl b	110 ± 6	**132 ± 3**	**139 ± 2**	112 ± 8
		Carotenoids	25 ± 2	29 ± 3	**40 ± 3**	33 ± 4
	leaf 7	*Chl a*	45 ± 4	**168 ± 4**	**188 ± 10**	64 ± 4
		Chl b	23 ± 3	**85 ± 3**	**92 ± 8**	33 ± 2
		Carotenoids	10.1 ± 0.8	**24.2 ± 0.8**	**28.6 ± 3.9**	12.4 ± 0.7
81 dpg	leaf 1	*Chl a*	96 ± 5	**128 ± 7**	**123 ± 7**	95 ± 5
		Chl b	55 ± 3	**67 ± 4**	**65 ± 4**	54 ± 3
		Carotenoids	19 ± 1	**24 ± 2**	**23 ± 2**	18 ± 1
	leaf 7	*Chl a*	10.9 ± 0.6	**52.3 ± 2.8**	**80.1 ± 4.3**	22.7 ± 1.2
		Chl b	5.7 ± 0.3	**27.3 ± 1.6**	**44.2 ± 2.6**	12.4 ± 0.7
		Carotenoids	3.3 ± 0.2	**11.2 ± 0.8**	**15.5 ± 1.0**	6.1 ± 0.4
**Number of nodes**			18 ± 0.3	19 ± 0.4	19 ± 0.3	17 ± 0,1
**Leaf FW (g m^{-2})**			302 ± 18	287 ± 5	283 ± 8	285 ± 10
**Leaf DW (g m^{-2})**			34 ± 4	34 ± 2	37 ± 2	41 ± 8
**Fruit number**			26 ± 2	29 ± 3	26 ± 1	28 ± 1
**Fruit FW (g)**			1.00 ± 0.16	0.82 ± 0.18	0.89 ± 0.09	1.05 ± 0.19
**Fruit DW (g)**			0.202 ± 0.007	0.182 ± 0.007	0.175 ± 0.006	0.191 ± 0.006
