# Supplementary material for: Expression of a Plastid-Targeted Flavodoxin Decreases Chloroplast Reactive Oxygen Species Accumulation and Delays Senescence in Aging Tobacco Leaves
Source: Front Plant Sci. 2018 Jul 17;9:1039. doi: 10.3389/fpls.2018.01039 (PMC6056745; doi:10.3389/fpls.2018.01039)
Supplement: Supplementary file 13 [file Table_2.PDF]

Supplementary Table S2. Protein identification by mass spectrometry (MS) from selected bands of Deriphat-PAGE. After electrophoretic separation (Figure 6), bands were excised from the gel and subjected to MALDI-TOF-TOF (see Materials and Methods). Proteins were identified through database search using the Mascot software. Band numbers correspond to those of the gel in Figure 6. Results were sorted for each band according to the Mascot score index. Accession numbers are from the NCBI database.

Accession No.	Description	Mascot Score	Mass (Da)	N° Matching peptides	Sequence coverage (%)	RMS error (ppm)
**Band No. 2**						
gi|115391901	photosystem I P700 chlorophyll a apoprotein A2 [Jasminum nudiflorum]	258	82478	10	17	40
gi|78102533	photosystem I P700 chlorophyll a apoprotein A1 [Nicotiana sylvestris]	177	82978	12	15	41
gi|225198	photosystem I P700 apoprotein A1	172	82938	11	13	41
gi|225198	photosystem I P700 apoprotein A1 (Nicotiana Tabacum)	148	82938	3	3	43
gi|11465984	photosystem II 47 kDa protein [Nicotiana tabacum]	148	55979	3	7	42
gi|169794099	photosystem II 47 kDa protein [Manihot esculenta]	237	56078	16	37	39
gi|81176277	photosystem II 47 kDa protein [Lactuca sativa]	237		16	37	39
gi|1027089237	PREDICTED: LOW QUALITY PROTEIN: photosystem II CP43 reaction center protein-like [Prunus mume]	158	89239	11	17	39
gi|1021511440	PREDICTED: photosystem II CP43 reaction center protein-like [Arachis ipaensis]	139	30845	7	24	43
gi|1012324807	Photosystem II CP43 chlorophyll apoprotein [Cajanus cajan]	147	14744	6	42	44
gi|332983528	photosystem II CP43 protein [Myricaria germanica]	145	16889	6	36	44
gi|51847683	PsbC [Ipomopsis aggregata]	144	15843	6	39	44
**Band No. 3**						
gi|11465954	photosystem I P700 chlorophyll a apoprotein A2 [Nicotiana tabacum]	332	82357	10	15	9
gi|914615637	photosystem I reaction center subunit II, chloroplastic [Nicotiana sylvestris]	178	22410	7	45	8
gi|697134314	PREDICTED: photosystem I reaction center subunit II, chloroplastic [Nicotiana tomentosiformis]	167	22639	6	37	8
gi|697134314	PREDICTED: photosystem I reaction center subunit II, chloroplastic [Nicotiana tomentosiformis]	70	22639	2	20	8
gi|78102533	photosystem I P700 chlorophyll a apoprotein A1 [Nicotiana sylvestris]	119	82978	9	11	8
gi|225198	photosystem I P700 apoprotein A1	67	82938	2	2	1
**Band No. 6**						
gi|1025054220	PREDICTED: chlorophyll a-b binding protein 50, chloroplastic-like, partial [Nicotiana tabacum]	200	22301	10	45	13
gi|1025057608	PREDICTED: chlorophyll a-b binding protein 40, chloroplastic-like [Nicotiana tabacum]	187	28275	9	35	15
gi|1025054216	PREDICTED: chlorophyll a-b binding protein 40, chloroplastic-like, partial [Nicotiana tabacum]	183	23211	8	35	14
gi|1025057612	PREDICTED: chlorophyll a-b binding protein 40, chloroplastic-like, partial [Nicotiana tabacum]	183	23324	8	34	14
gi|1025051919	PREDICTED: chlorophyll a-b binding protein 16, chloroplastic-like [Nicotiana tabacum]	184	24966	9	40	15
gi|1025406480	PREDICTED: chlorophyll a-b binding protein 16, chloroplastic [Nicotiana tabacum]	187	28160	9	35	15
gi|404474510	ATP synthase CF1 alpha subunit (chloroplast) [Capsicum annuum]	168	55458	13	29	21
gi|78102516	ATP synthase CF1 alpha subunit [Nicotiana sylvestris]	174	55388	13	29	18
gi|11769	alpha subunit of ATPase [Nicotiana tabacum]	166	55404	12	26	18
gi|81301547	ATP synthase CF1 alpha subunit [Nicotiana tomentosiformis]	159	55344	11	23	19
gi|11769	alpha subunit of ATPase [Nicotiana tabacum]	80	55404	2	5	11
**Band No. 7**						
gi|1027859155	oxygen-evolving enhancer protein 3-2, chloroplastic-like [Nicotiana tabacum]	136	24142	9	42	17
gi|1027858090	chlorophyll a-b binding protein CP26, chloroplastic [Nicotiana tabacum]	112	30428	10	47	14
gi|396261	23 kDa polypeptide of water-oxidizing complex of photosystem II [Nicotiana tabacum]	101	21958	4	40	12
gi|1027856696	oxygen-evolving enhancer protein 2-3, chloroplastic [Nicotiana tabacum]	99	28666	5	27	19
gi|1027856706	chlorophyll a-b binding protein CP29.1, chloroplastic-like [Nicotiana tabacum]	98	31132	7	23	22
gi|1025054220	PREDICTED: chlorophyll a-b binding protein 50, chloroplastic-like, partial [Nicotiana tabacum]	96	22301	7	37	15
gi|1025051919	PREDICTED: chlorophyll a-b binding protein 16, chloroplastic-like [Nicotiana tabacum]	93	24966	7	32	16
gi|1027855844	oxygen-evolving enhancer protein 2-2, chloroplastic [Nicotiana tabacum]	93	28543	3	19	6
gi|1025057612	PREDICTED: chlorophyll a-b binding protein 40, chloroplastic-like, partial [Nicotiana tabacum]	92	23324	7	33	18
gi|1025054216	PREDICTED: chlorophyll a-b binding protein 40, chloroplastic-like, partial [Nicotiana tabacum]	92	23211	6	33	16
gi|1025406480	PREDICTED: chlorophyll a-b binding protein 16, chloroplastic [Nicotiana tabacum]	91	28160	6	28	12
gi|1025057608	PREDICTED: chlorophyll a-b binding protein 40, chloroplastic-like [Nicotiana tabacum]	91	28275	6	28	12
**Band No. 9**						
gi|720025074	PREDICTED: oxygen-evolving enhancer protein 1, chloroplastic [Nelumbo nucifera]	121	35242	7	28	23
gi|1025054220	PREDICTED: chlorophyll a-b binding protein 50, chloroplastic-like, partial [Nicotiana tabacum]	115	22301	3	19	13
gi|1025054216	PREDICTED: chlorophyll a-b binding protein 40, chloroplastic-like, partial [Nicotiana tabacum]	106	23211	2	10	13
gi|1025057612	PREDICTED: chlorophyll a-b binding protein 40, chloroplastic-like, partial [Nicotiana tabacum]	106	23324	2	10	13
gi|1025406480	PREDICTED: chlorophyll a-b binding protein 16, chloroplastic [Nicotiana tabacum]	114	28160	3	13	14
gi|1025051919	PREDICTED: chlorophyll a-b binding protein 16, chloroplastic-like [Nicotiana tabacum]	106	24966	2	10	13
**Band No. 10**						
gi|332983528	photosystem II CP43 protein [Myricaria germanica]	273	16889	9	50	12
gi|922560315	PREDICTED: photosystem II CP43 reaction center protein-like [Brassica oleracea var. oleracea]	260	30661	10	33	11
gi|1003360103	PSII 43 kDa protein, partial (chloroplast) [Panax ginseng]	254	28668	9	29	12
gi|1002161119	photosystem II 44 kDa protein (chloroplast) [Tetrastigma hemsleyanum]	251	52150	11	21	13
gi|13625479	photosystem II 44 kDa protein [Nicotiana tabacum]	167	50313	4	13	13
gi|1025162842	PREDICTED: chlorophyll a-b binding protein 7, chloroplastic [Nicotiana tabacum]	172	28317	9	29	15
gi|922428637	PREDICTED: chlorophyll a-b binding protein CP29.1, chloroplastic [Brassica oleracea var. oleracea]	104	31253	6	22	27
gi|698438113	PREDICTED: chlorophyll a-b binding protein CP29.1, chloroplastic-like [Nicotiana sylvestris]	90	31033	4	16	16
gi|1027856706	chlorophyll a-b binding protein CP29.1, chloroplastic-like [Nicotiana tabacum]	90	31132	4	16	16
gi|1027856706	chlorophyll a-b binding protein CP29.1, chloroplastic-like [Nicotiana tabacum]	71	31132	2	8	11
