# Supplementary material for: Expression of a Plastid-Targeted Flavodoxin Decreases Chloroplast Reactive Oxygen Species Accumulation and Delays Senescence in Aging Tobacco Leaves
Source: Front Plant Sci. 2018 Jul 17;9:1039. doi: 10.3389/fpls.2018.01039 (PMC6056745; doi:10.3389/fpls.2018.01039)
Supplement: Supplementary file 14 [file Table_3.PDF]

Supplementary Table S3. Metabolite levels in leaves 1 and 7 of WT, *pfld* and *cfld* plants. Extracts were prepared from leaves 1 and 7 at 73 dpg, and metabolite contents were determined as described in Materials and Methods. Values are the means ± SE of 3-5 biological replicates. Significant differences (ANOVA, $P < 0.05$) between transgenic and WT plants are shown in bold.

	leaf 1				leaf 7			
**Metabolite, nmol g^{-1} FW**	**WT**	***pfld*5-8**	***pfld*4-2**	***cfld*1-4**	**WT**	***pfld*5-8**	***pfld*4-2**	***cfld*1-4**
**3-P-glycerate**	139 ± 23	166 ± 29	174 ± 21	143 ± 19	81.3 ± 12.5	**123 ± 16**	**121 ± 15**	69.1 ± 9.3
**ADP-Glc**	0.49 ± 0.10	0.34 ± 0.04	0.69 ± 0.13	0.45 ± 0.09	0.39 ± 0.05	0.59 ± 0.15	0.50 ± 0.09	0.30 ± 0.05
**UDP-Glc**	17.7 ± 2.0	**37.1 ± 5.0**	**36.3 ± 4.5**	20.3 ± 2.5	31.7 ± 4.3	**54.9 ± 7.5**	**55.4 ± 7.7**	31.9 ± 4.3
**P-*enol*-pyruvate**	59.5 ± 8.1	**697 ± 286**	**2274 ± 1034**	72.6 ± 23.2	25.1 ± 11.4	**426 ± 168**	**333 ± 133**	27.1 ± 5.1
**Fru-6-P**	6.37 ± 0.76	**11.6 ± 0.6**	**12.0 ± 0.6**	6.87 ± 0.87	7.65 ± 0.49	**11.0 ± 0.5**	**11.3 ± 0.6**	7.12 ± 0.32
**Glc-6-P**	22.5 ± 1.4	**29.8 ± 1.8**	**32.7 ± 2.5**	27.0 ± 1.5	21.2 ± 1.8	**46.3 ± 3.2**	**38.4 ± 1.9**	21.1 ± 1.8
**Suc-6-P**	1.31 ± 0.16	**2.34 ± 0.43**	**2.06 ± 0.41**	1.61 ± 0.17	1.13 ± 0.10	**2.15 ± 0.25**	**2.04 ± 0.23**	1.16 ± 0.11
***cis*-Aconitate**	2.73 ± 0.32	3.24 ± 0.25	2.93 ± 0.29	2.56 ± 0.32	4.94 ± 0.53	**3.41 ± 0.39**	**2.75 ± 0.31**	5.29 ± 0.62
**Citrate**	395 ± 66	**1014 ± 187**	**638 ± 75**	339 ± 38	723 ± 142	860 ± 115	816 ± 259	445 ± 51
**Fumarate**	1.14 ± 0.09	1.38 ± 0.08	1.36 ± 0.10	1.19 ± 0.07	11.16 ± 0.99	**3.62 ± 0.31**	**3.63 ± 0.26**	8.62 ± 0.59
**Isocitrate**	402 ± 57	**1000 ± 51**	**644 ± 79**	340 ± 27	1039 ± 263	1028 ± 150	1075 ± 288	509 ± 54
**Malate**	1125 ± 124	1318 ± 90	1239 ± 122	733 ± 63	3250 ± 138	**4047 ± 179**	**3950 ± 151**	2872 ± 76
**2-Oxoglutarate**	87.5 ± 9.3	94.6 ± 9.8	93.9 ± 11.3	69.4 ± 4.8	149.6 ± 8.9	**256.1 ± 24.1**	**186.9 ± 12.7**	145.5 ± 15.2
**Succinate**	4.64 ± 0.35	5.09 ± 0.32	5.91 ± 0.64	5.31 ± 0.53	2.15 ± 0.21	**1.56 ± 0.13**	**1.45 ± 0.09**	2.40 ± 0.19
***trans*-Aconitate**	0.38 ± 0.05	0.61 ± 0.08	0.54 ± 0.10	0.40 ± 0.04	0.65 ± 0.05	0.53 ± 0.06	0.48 ± 0.06	0.59 ± 0.08
**μmol g^{-1} FW**								
**Glc**	4.65 ± 0.66	5.14 ± 0.79	7.07 ± 1.18	5.49 ± 0.69	27.5 ± 2.0	**16.9 ± 2.4**	**19.3 ± 1.6**	29.6 ± 1.6
**Fru**	2.50 ± 0.58	4.10 ± 0.59	**5.39 ± 0.66**	3.72 ± 0.49	15.9 ± 1.7	**7.8 ± 0.9**	**9.3 ± 0.8**	18.0 ± 1.4
**Glc + Fru**	7.15 ± 1.21	9.23 ± 1.34	12.46 ± 1.75	9.21 ± 1.15	43.4 ± 3.6	**24.6 ± 3.3**	**28.6 ± 2.3**	47.5 ± 2.8
**Suc**	17.6 ± 0.8	**23.0 ± 1.8**	20.8 ± 1.1	20.0 ± 1.2	2.59 ± 0.18	**5.67 ± 0.31**	**4.54 ± 0.31**	2.73 ± 0.19
**Starch**	107.5 ± 3.3	**53.9 ± 5.4**	**40.8 ± 3.1**	105.5 ± 2.9	58.8 ± 7.5	**30.9 ± 5.2**	**20.8 ± 3.2**	45.2 ± 5.9
**Starch/ Suc**	6.23 ± 0.35	**2.37 ± 0.17**	**2.01 ± 0.18**	5.42 ± 0.31	23.8 ± 3.1	**5.44 ± 0.83**	**4.56 ± 0.60**	16.3 ± 1.53
**(Glc + Fru)/Suc**	0.41 ± 0.06	0.39 ± 0.05	0.59 ± 0.07	0.46 ± 0.05	17.8 ± 2.0	**4.46 ± 0.69**	**6.71 ± 0.87**	18.5 ± 2.0
**(Glc + Fru +Suc)/Starch**	0.232 ± 0.016	**0.630 ± 0.071**	**0.845 ± 0.070**	0.277 ± 0.019	0.93 ± 0.15	1.07 ± 0.26	1.70 ± 0.27	1.39 ± 0.26
