# Supplementary material for: Expression of a Plastid-Targeted Flavodoxin Decreases Chloroplast Reactive Oxygen Species Accumulation and Delays Senescence in Aging Tobacco Leaves
Source: Front Plant Sci. 2018 Jul 17;9:1039. doi: 10.3389/fpls.2018.01039 (PMC6056745; doi:10.3389/fpls.2018.01039)
Supplement: Supplementary file 15 [file Table_4.PDF]

Supplementary Table S4. Amino acid levels in leaves 1 and 7 of WT, *pfld* and *cfld* plants. Extracts were prepared from leaves 1 and 7 at 73 dpg, and amino acid contents were determined as described in Materials and Methods. Values are the means ± SE of 6-10 independent plants from each genotype. Significant differences (ANOVA, P < 0.05) between transgenic and WT plants are shown in bold.

	leaf 1				leaf 7			
**Aminoacid, nmol g^{-1} FW**	**WT**	***pfld* 5-8**	***pfld* 4-2**	***cfld* 1-4**	**WT**	***pfld* 5-8**	***pfld* 4-2**	***cfld* 1-4**
**His**	69.3 ± 5.9	113.8 ± 14.4	**110.0 ± 6.5**	69.8 ± 3.6	18.8 ± 2.6	27.2 ± 4.6	27.2 ± 4.6	16.7 ± 2.2
**Asn**	111 ± 7	145 ± 17	141 ± 8	**152 ± 9**	51.5 ± 3.3	59.5 ± 6.3	71.0 ± 5.9	53.6 ± 6.5
**Ser**	470 ± 39	669 ± 99	717 ± 85	945 ± 107	140 ± 11	**247 ± 34**	**255 ± 25**	123 ± 11
**Gln**	118 ± 19	**225 ± 50**	**212 ± 17**	**230 ± 15**	230 ± 29	271 ± 26	270 ± 29	258 ± 35
**Gly**	247 ± 16	278 ± 40	255 ± 23	263 ± 17	127 ± 10	**187 ± 27**	**186 ± 23**	108 ± 12
**Asp**	425 ± 45	737 ± 106	549 ± 66	533 ± 52	46.5 ± 5.7	**135 ± 22**	**123 ± 22**	48.5 ± 12.4
**Glu**	4069 ± 341	5686 ± 711	5428 ± 503	4868 ± 164	901 ± 132	**1980 ± 328**	**2052 ± 273**	907 ± 110
**Thr**	429 ± 34	**1043 ± 248**	**1066 ± 78**	500 ± 18	97.4 ± 7.1	**194 ± 25**	**199 ± 26**	86.0 ± 7.5
**Ala**	443 ± 43	678 ± 101	647 ± 47	540 ± 19	138 ± 12	**242 ± 25**	**271 ± 30**	127 ± 12
**γ-aminobutiric acid**	389 ± 48	541 ± 99	566 ± 66	488 ± 35	335 ± 30	**499 ± 47**	**506 ± 61**	269 ± 33
**Pro**	480 ± 42	**1564 ± 237**	**1387 ± 114**	641 ± 26	111 ± 10	**351 ± 47**	**366 ± 41**	113 ± 10
**Lys**	55.4 ± 6.2	**105.3 ± 15.7**	**103.1 ± 6.2**	75.8 ± 3.4	29.7 ± 3.8	**41.1 ± 4.0**	**44.4 ± 4.0**	31.3 ± 2.4
**Val**	208 ± 15	267 ± 35	240 ± 20	234 ± 10	57.3 ± 5.5	**87.9 ± 12.7**	**91.3 ± 7.9**	51.5 ± 4.5
**Ile**	128 ± 9	191 ± 20	195 ± 14	149 ± 6	37.5 ± 3.7	**68.6 ± 9.4**	**62.4 ± 5.2**	34.5 ± 3.1
**Leu**	163 ± 12	**270 ± 39**	**266 ± 28**	180 ± 11	51.5 ± 4.6	**73.3 ± 9.5**	**72.5 ± 6.2**	48.1 ± 4.2
**Phe**	96.4 ± 6.7	**141 ± 12**	**146 ± 12**	105 ± 3	44.1 ± 3.8	52.4 ± 6.9	54.1 ± 3.7	44.8 ± 4.2
**Gln/Glu**	0.029 ± 0.004	0.036 ± 0.006	0.036 ± 0.003	**0.048 ± 0.004**	0.295 ± 0.041	**0.150 ± 0.022**	**0.143 ± 0.018**	0.301 ± 0.042
**Asn/Asp**	0.281 ± 0.024	0.210 ± 0.014	0.291 ± 0.027	0.285 ± 0.041	1.22 ± 0.14	**0.58 ± 0.09**	**0.65 ± 0.08**	1.32 ± 0.33
**Glu + Asp/Gln + Asn**	18.8 ± 1.3	23.7 ± 3.2	20.8 ± 2.0	16.1 ± 1.3	3.24 ± 0.51	**6.74 ± 0.99**	**6.73 ± 0.85**	3.52 ± 0.68
**Gly/Ser**	0.557 ± 0.030	**0.432 ± 0.032**	**0.371 ± 0.025**	**0.322 ± 0.047**	0.919 ± 0.062	**0.764 ± 0.047**	**0.720 ± 0.034**	0.857 ± 0.043
