# Supplementary material for: Expression of a Plastid-Targeted Flavodoxin Decreases Chloroplast Reactive Oxygen Species Accumulation and Delays Senescence in Aging Tobacco Leaves
Source: Front Plant Sci. 2018 Jul 17;9:1039. doi: 10.3389/fpls.2018.01039 (PMC6056745; doi:10.3389/fpls.2018.01039)
Supplement: Supplementary file 16 [file Table_5.PDF]

Supplementary Table S5. Levels of cytokinins and auxins in WT, *pfld* and *cfld* leaves. Extracts were prepared from leaves 1 and 7 at 73 dpg, and hormone contents were determined as described in Materials and Methods. Values are the means ± SE of 3-5 biological replicates. Significant differences (ANOVA, P < 0.05) between transgenic and WT plants are shown in bold.

	leaf 1				leaf 7			
**Metabolite, nmol g^{-1} FW**	**WT**	***pfld* 5-8**	***pfld* 4-2**	***cfld* 1-4**	**WT**	***pfld* 5-8**	***pfld* 4-2**	***cfld* 1-4**
**Auxins**								
**Indole-3-Acetamide**	1.31 ± 0.19	**2.25 ± 0.65**	1.87 ± 0.27	1.18 ± 0.25	0.914 ± 0.174	1.454 ± 0.255	1.171 ± 0.277	0.500 ± 0.136
**Indole-3-Acetic Acid**	10.9 ± 0.5	11.9 ± 1.3	9.7 ± 0.6	11.7 ± 0.6	5.45 ± 0.22	8.10 ± 1.51	8.05 ± 1.05	3.93 ± 0.36
**Indole-3-Acetyl-L-Alanine**	0.812 ± 0.064	**1.314 ± 0.042**	0.955 ± 0.101	0.689 ± 0.094	0.558 ± 0.062	0.657 ± 0.084	0.749 ± 0.116	0.673 ± 0.086
**Oxindole-3-Acetic Acid**	228 ± 15	**68 ± 10**	**87 ± 7**	**158 ± 15**	25 ± 8	48 ± 14	47 ± 9	37 ± 9
**Citokinins**								
***cis*-Zeatin riboside**	0.043 ± 0.006	**0.076 ± 0.023**	**0.098 ± 0.014**	0.044 ± 0.004	0.058 ± 0.008	**0.097 ± 0.013**	0.080 ± 0.007	0.076 ± 0.009
***cis*-Zeatin**	0.048 ± 0.004	0.093 ± 0.026	0.082 ± 0.015	0.064 ± 0.012	0.043 ± 0.035	0.077 ± 0.024	0.103 ± 0.011	0.063 ± 0.015
**Dihydrozeatinriboside**	0.274 ± 0.052	0.164 ± 0.043	0.180 ± 0.031	0.221 ± 0.051	0.994 ± 0.093	**0.572 ± 0.083**	**0.634 ± 0.054**	**1.826 ± 0.322**
**N6-Isopentenyladenosine riboside**	0.065 ± 0.013	**0.167 ± 0.048**	**0.162 ± 0.018**	0.062 ± 0.010	0.071 ± 0.024	**0.225 ± 0.022**	**0.161 ± 0.023**	0.084 ± 0.015
