# Supplementary material for: Expression of a Plastid-Targeted Flavodoxin Decreases Chloroplast Reactive Oxygen Species Accumulation and Delays Senescence in Aging Tobacco Leaves
Source: Front Plant Sci. 2018 Jul 17;9:1039. doi: 10.3389/fpls.2018.01039 (PMC6056745; doi:10.3389/fpls.2018.01039)
Supplement: Supplementary file 17 [file Table_6.PDF]

Supplementary Table S6. Primers used for qRT-PCR.

Gene	Accession number	Sequence	Tm (°C)	Product length (pb)
N. tabacum cysteine protease (*SAG-12*)	HQ108340.1	5'-TCCATACAAGGGAGAAGATG -3'	55.1	146
		5'-CTATTGCCACCGAAACAG -3'	55.1	
Putative *N. tabacum* "stay-green" (*SGR*) protein	EU294209.1	5'-GGTGGCAAAATGAGAAATAC -3'	54.7	126
		5'-AATATCATCAGAAGGCCAAG -3'	54.4	
Putative *N. tabacum* "chloroplast vesiculation" (*CV*) protein	FG644289.1	5'-TGGGAACTCTTTAGAAACCA-3'	54.9	122
		5'-ATCTTGCCACCGACTTTAC -3'	55.2	
N. tabacum elongation factor 1-α (*EF-1α*)	AF120093.1	5'-TTCAGGAGCATGCGTCAAACTG-3'	55.9	100
		5'-TCTTCTTCTGAGCAGCCTTGGT-3'	55.6	
